# Sustainable Access to π-Conjugated Molecular Materials via Direct (Hetero)Arylation Reactions in Water and under Air

**DOI:** 10.3390/molecules25163717

**Published:** 2020-08-14

**Authors:** Adiel Mauro Calascibetta, Sara Mattiello, Alessandro Sanzone, Irene Facchinetti, Mauro Sassi, Luca Beverina

**Affiliations:** 1 Department of Materials Science, University of Milano-Bicocca, Via R. Cozzi, 55, I-20125 Milano, Italy; a.calascibetta@campus.unimib.it (A.M.C.); a.sanzone@campus.unimib.it (A.S.); i.facchinetti3@campus.unimib.it (I.F.); 2 Department of Materials Science, University of Milano-Bicocca and INSTM, Via R. Cozzi, 55, I-20125 Milano, Italy; sara.mattiello@unimib.it (S.M.); mauro.sassi@unimib.it (M.S.)

**Keywords:** direct arylation, surfactants, emulsions, organic semiconductors

## Abstract

Direct (hetero)arylation (DHA) is playing a key role in improving the efficiency and atom economy of C–C cross coupling reactions, so has impacts in pharmaceutical and materials chemistry. Current research focuses on further improving the generality, efficiency and selectivity of the method through careful tuning of the reaction conditions and the catalytic system. Comparatively fewer studies are dedicated to the replacement of the high-boiling-point organic solvents dominating the field and affecting the overall sustainability of the method. We show herein that the use of a 9:1 *v*/*v* emulsion of an aqueous Kolliphor 2 wt% solution while having toluene as the reaction medium enables the preparation of relevant examples of thiophene-containing π-conjugated building blocks in high yield and purity.

## 1. Introduction

Solution-processable π-conjugated organic materials are key components of a vast array of applications ranging from photonics to (opto)electronics [1,2,3,4,5,6]. New materials are reported daily, constantly updating the record performances in the respective application fields. Comparatively fewer contributions are dedicated to the transition from proof of concept laboratory synthesis and characterization to the industrial environment. Aside from improved stability and processability, one of the main issues still hampering full industrial exploitation is the overall sustainability of the synthetic protocols [7,8,9]. Researchers working in the field of printed (opto)electronics are becoming increasingly aware of the need for materials combining reasonable performances with sustainable synthetic access [10,11]. Such an endeavor requires a certain redirection in the design criteria guiding the development of new materials and a drastic improvement of the synthetic methodologies employed [12,13].

Carbon–carbon bond forming reactions are the cornerstone of the synthesis of organic π-conjugated derivatives. Amongst them, the Stille and Suzuki–Miyaura (S–M) couplings have a dominating position for both small molecules and polymers [14,15,16,17]. From the standpoint of environmental concerns, the Stille reaction is particularly problematic due to the formation of toxic alkylstannanes in stoichiometric amounts. The S–M coupling poses no intrinsic toxicity concerns and offers high tolerance to functional groups, but shares with the Stille coupling the need for the activation of one of the two reactants, in this case in the form of a boronic acid, ester or trifluoroborate. Palladium-catalyzed direct (hetero)arylation (DHA) is a recent and rapidly improving method enabling the direct coupling of an (hetero)aryl and an (hetero)aryl halide without the need for activation of the former [18,19,20]. The advantages in terms of atom economy and the number of synthetic steps required are obvious. Formerly limited in terms of selectivity and generality, the method is now seriously competing with Stille and S–M in particular for the manufacturing of thiophene-containing molecules and polymers [21,22,23,24,25,26]. Careful tuning of reaction conditions enables selective arylation at the 2-position of the thiophene ring with good tolerance for a wide range of electron-donating and electron-withdrawing groups. Due to the pervasive use of thiophene derivatives in materials for organic optoelectronics, DHA is becoming a prominent tool in the field [20,21,27,28]. Leclerc, Luscombe and Sommer, amongst others, are further expanding the scope of the reaction by developing DHA polymerization (DHAP). The method possesses generality and leads to high molecular weights, low defects concentration and good regioregularity [29,30,31].

A thorough mechanistic understanding of the reaction is still lacking, but there is a consensus on a base-assisted, concerted metalation-deprotonation (CMD) pathway [18,24,32,33,34]. It is also generally observed that the presence of a sterically hindered carboxylic acid (pivalic acid in particular) helps the reaction [35,36]. The latter coordinates with the metal center in situ and assists in the deprotonation transition state. Apart from the need for basic conditions and for a carboxylate additive, the details of the various literature protocols vary significantly in terms of palladium source, phosphine, solvent and duration [18,37]. The preferred solvents are high-boiling-point aromatic derivatives, chlorobenzene and toluene in particular, as the reaction requires prolonged heating at high temperatures: a relative weakness of the protocol. In fact, high-boiling-point aromatic solvents are difficult to recycle and generate wastes. The atom economy metric accounts only for the efficient use of atoms contained in the reagents but disregards the solvent. The overall evaluation of the sustainability of a process also requires the evaluation of the amounts of solvents employed, through a metric defined as the E-factor of the reaction. The latter is the mass ratio between organic wastes and product and typically ranges from 10 for specialty chemicals to a few hundred for complex drugs [38]. The syntheses of organic semiconductors, both molecular and polymeric, are still far above such reference values.

Replacing the aromatic solvent with water would be the obvious solution. In this respect, the general hydrophobicity of organics does not represent an immediate obstacle. A constantly increasing body of work developed at universities and research institutes provides convincing evidence that a surprisingly vast number of reactions on organics can be performed in water, provided that a suitable surfactant is present [39,40]. The role played by the surfactant is the creation of lipophilic domains where organics can accumulate in high concentrations and efficiently react. The details of the colloidal state best describing the reaction at all stages depend upon the natures and concentrations of reagents and products, yet there is little doubt that surfactant-enhanced reactions have impressive generality, high efficiency and unsurpassed sustainability due to the critical reduction of the organic waste produced [41]. Such methods already contributed to the green chemistry-compliant access to relevant organic-conjugated derivatives [42,43,44,45,46]. In particular, the use of the industrial grade, FDA-approved surfactant Kolliphor EL (K-EL)—a complex mixture obtained by the reaction of castor oil with 35 equivalents of ethylene oxide—in S–M and Buchwald–Hartwig (B–H) amination protocols, enabled the high yield and low E-factor preparation of several classes of molecular semiconductors, including perylenediimides, isoindigos, oligothiophenes and benzothiadiazoles, having applications in organic field effect transistors, organic solar cells and luminescent solar concentrators [47].

The extension of the use of aqueous media to DHA reactions would seem natural, yet the literature so far reports only very few examples limited to substituted biaryls and requiring the assistance of directing groups and the use of an inert atmosphere [48,49,50]. The group of M. Leclerc [51,52] recently approached water as a medium: they reported that the use of a water/toluene 1:1 vol/vol biphasic mixture enables a sizeable improvement in the molecular weights of a series of thiophene-containing polymers over more standard homogeneous phase conditions. As a further benefit, they demonstrated that the reaction is oxygen insensitive. Albeit impressive, such results are not relevant in terms of E-factor reduction, as the amount of organic solvent employed is the same in homogeneous and biphasic reactions. Due to being inspired and—thanks to such seminal results—aware of the compatibility of DHA with the presence of both water and oxygen, we here show the first study of efficient surfactant enhanced direct (hetero)arylation reactions, carried out in water and under air with a stoichiometric amount of organic solvent. Our method enables the preparation of a large variety of thiophene-flanked π-conjugated building blocks. We demonstrate high efficiency along with E-factors in the 10^2^ order, including purification.

## 2. Results

### 2.1. Optimization of Surfactant Enhanced DHA in Water on a Model Reaction

The aim of this work is the replacement with water of the organic solvent in DHA reactions. In our previous experience with S-M couplings, we profitably exploited a 2 wt% K-EL solution as an ideal reaction medium to drive the reaction to completion in a short time, at a low temperature and with a high yield, even working under an oxygenated environment [42,43,44,45,46]. The extension of such an approach to DHA is not possible due to a fundamental property of micellar solutions: neutral surfactants (such as K-EL and the popular TPGS-750-M, a pegylated derivative of tocopherol) [53] all possess a characteristic temperature defined as the cloud point, above which thermally induced demixing is observed. The cloud point is pH dependent and under the basic conditions required by DHA generally does not exceed 40–50 °C [54]. Since high temperatures are normally required to activate (hetero)aromatic C–H bonds (T ≥ 100 °C), the use of a micellar solution becomes impossible, so we had to revert to oil-in-water emulsions with a minimum amount of an organic solvent. As the opposite with respect to micellization, emulsification is a thermally promoted phenomenon compatible with the characteristic conditions of the DHA. The approach has a certain generality and is usually referred to as the co-solvent approach in the micellar catalysis literature [55].

Analogously to our previous work, we selected the mixture of K-EL 2 wt% in water with toluene 9:1 *v*/*v* as the preferred reaction medium [43]. The amount of organic solvent involved in such reactions is really minimal. Indeed, the molarity of toluene is 0.13, to be compared with the 0.5 formal molarity of the halide to be arylated. Toluene helps with making the reaction possible but does not act as a solvent—rather, as a stabilizer for the emulsion.

**Scheme 1:**
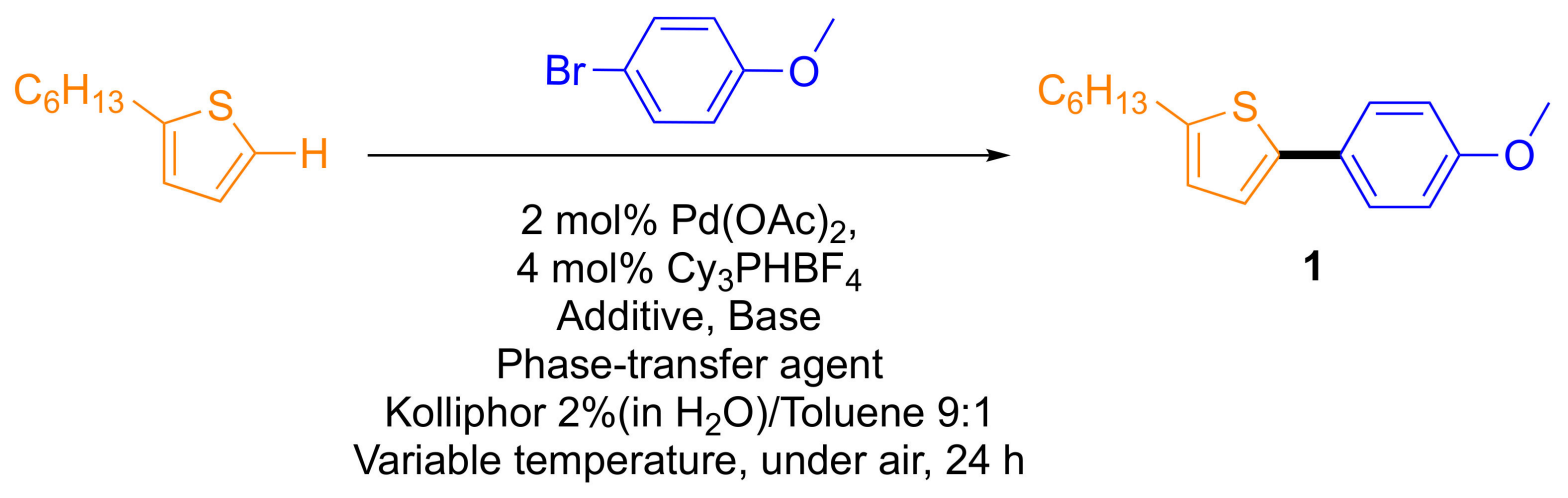
Direct arylation of 2-hexylthiophene with 4-bromoanisole.

We chose the arylation of 2-hexylthiophene with 4-bromoanisole as the model reaction (Scheme 1). The choice was motivated by the vast amount of DHA literature on both thiophene derivatives as reactants and 4-bromoanisole as the arylating species [56].

Surfactant-enhanced reactions involve the formation of a micro-heterogeneous environment wherein the partition coefficient of the species between the oil and water phase is a key factor in achieving high efficiency. Indeed, the fact that all reagents and catalysts are present at the same time in the same flask does not necessarily imply that they can also localize within the same phase in the medium. We thus carried out a detailed optimization of the reaction conditions, paying particular attention to colocalization of the various reactive species within the lipophilic pockets where both halide and aryl are more likely to localize (Table 1).

We focused our attention on the variation of the carboxylic acid additive, the nature of the base, the presence of a phase-transfer agent and the temperature. We kept the catalytic system fixed, using the popular and affordable combination of palladium acetate (Pd(OAc)_2_, 2 mol%) and the bulky tricyclohexylphosphine (PCy_3_), generated in situ by the deprotonation of corresponding air stable tetrafluoroborate salt Cy_3_PHBF_4_ (4 mol%). Such a catalytic system is particularly performant in reactions carried out in toluene and xylene mixtures [57,58,59]. As the baseline experiment, we used literature DHA conditions giving good results when using toluene as the solvent: the presence of pivalic acid (PivOH, 30 mol%) as the proton shuttle assistant between the base and the catalytic species and Na_2_CO_3_ (1.5 eq) as the base. As is shown in Table 1 entry 1, performing the reaction at 80 °C leads to negligible conversion. The product could be barely detected by the GC–MS analysis of the reaction mixture. In entries 2 and 3, we studied the effect of using pivalate salts of potassium (PivOK, entry 2) and tetrabutylammonium (PivONBu_4_, entry 3). The use of PivOK in particular as a direct source of carboxylate instead of PivOH/Na_2_CO_3_ in the presence of PCy_3_ as ligand, was already successfully employed by Sommer in DHAP [60]. Under our experimental conditions, conversion was minimal in both cases.

**Scheme 2:**
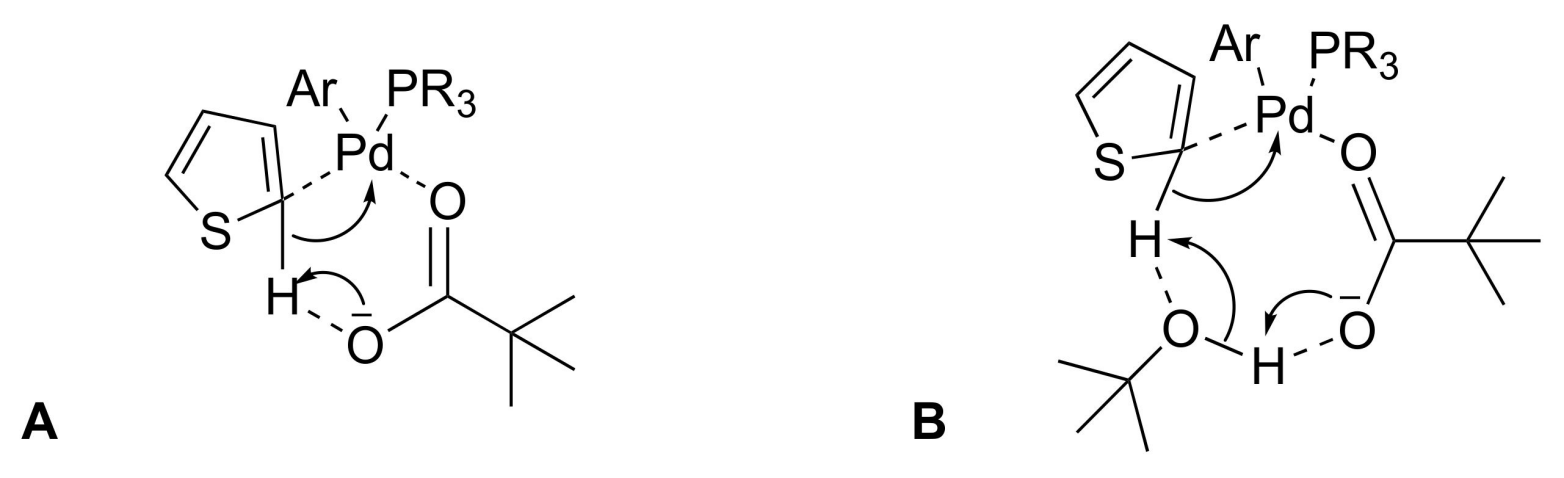
(**A**) Carboxylate assistance C–H bond activation/cleavage in CMD transition state. (**B**) Proposed C–H bond activation/cleavage transition state *tert*-butanol assisted in CMD mechanism.

The increase of the temperature from 80 to 130 °C (reactions performed in a pressure tight vessel) gave sizably improved results. Entry 4 shows that working under the very same conditions as entry 1 (apart from the temperature), the arylation product is formed in 30% yield. The following optimization steps were based on the colocalization of reactive species we previously mentioned. 2-Hexylthiophene, 4-bromoanisole and the palladium complex formed in situ by the reaction of Pd(OAc)_2_ with PCy_3_ have a lipophilic character. Conversely, the base and the carboxylate salt are both hydrophilic. Leclerc, while working with a water/toluene biphasic mixture, obtained satisfactory results only by working with an essentially saturated water solution of carbonate and driving the reaction at the interphase [51,52]. Such a solution was unpractical in our case as the amount of toluene was so little that an interfacial reaction have would been exceedingly slow. Instead, we tried to improve the repartition of the base between the different domains of our micro-heterogeneous environment. Entry 5 shows that the exchange of Na_2_CO_3_ with Cs_2_CO_3_ has a negligible effect. Truly, the latter is somewhat soluble in polar organic solvents but remains essentially insoluble in toluene. Conversely, the introduction of a 30 mol% amount of the cationic surfactant Aliquat 336 (a mixture of quaternary chloride ammonium salts; it contains a mixture of C_8_ (octyl) and C_10_ (decyl) chains with C_8_ predominating) in connection with Na_2_CO_3_ (entry 6) sizably improves the reaction, raising the yield from the 30% of entry 4 to 53%. We believe that the Aliquat 336 acts as a phase transfer agent for the base, thereby improving the repartition of the carbonate between the different phases. Tetra alkylammonium salts are not particularly stable at high temperatures and in an alkaline environment; we thus exchanged the Aliquat 336 with Aliquat HTA-1, a guanidinium salt specifically developed for high temperature applications (the detailed structure of said surfactant is undisclosed by the provider). The yield of the reaction was only marginally improved (entry 7) on going from 53% to 55%, but purification was easier. Next, we increased the strength of the base by replacing Na_2_CO_3_ with NaOH (entry 8) and sodium *tert*-butoxide (*t*BuONa, entry 9). When working in water, the two bases were identical and the real difference between entry 8 and 9 was the introduction of *tert*-butanol (*t*BuOH). If the use of NaOH alone gave 48% yield, slightly lower than that observed with Na_2_CO_3_ (55%), the introduction of *t*BuOH improved the yield to 59%. The result could be explained by the impact of *t*BuOH on the reactive formulation. Said alcohol is a hydrotrope—that is, a small molecule that does not spontaneously form micelles in water solutions but assists the formation of emulsions when a lyophobic phase is introduced in an aqueous solution. In formulation chemistry, hydrotropes are effectively used as cosurfactants for stabilization of microemulsions by reducing the interfacial tension between water and oil [61]. Moreover, the possibility of heteroaryl C–H bond activation/cleavage transition state assistance from *tert*-butanol in CMD mechanism, by means of supramolecular H-bonds interactions, should not be excluded (Scheme 2). The energy necessary to attain the transition state involved in the rate determining step of DHA can be decomposed into two contributions [24,32,33,34]. The first corresponds to the energy necessary to distort the C–H bond (E_dist_) and the second to the energy necessary to counter the electronic interaction between the distorted substrate and the metal center (E_int_). The transition state proposed (Scheme 2B) may involve a supramolecular distortion of the eight-membered ring via hydrogen bonds, in which the *tert*-butanol inserts between the activated C–H bond and the carboxylate ligand. From an energetic point of view this should lead to a raised E_dist_ for the increased tension derived by *t*BuOH insertion. By such interactions, the *tert*-butanol can swiftly transfer its acid proton to the carboxylate ligand. Accordingly, the *tert*-butoxide transient specie formed, which is a stronger Lewis base compared to the carboxylate with delocalized electrons, can cleave the activated heteroaromatic C–H bond with a faster proton transfer, reducing the transition energy cost E_int_ in the CMD process.

The real breakthrough in the optimization of the method came with the introduction of neodecanoic acid (NDA, a mixture of carboxylic acids with the common structural formula C_10_H_20_O_2_, a molecular weight of 172.26 g/mol, and the CAS number 26896-20-8. Components of the mixture are acids with the common property of a “trialkyl acetic acid” having three alkyl groups at carbon two) in the place of pivalic acid. NDA possesses a lipophilic character (logP = 3.6) much more pronounced than that of pivalic acid (logP = 1.5). Besides, the use of bulkier NDA instead of PivOH in DHA reactions is reported to improve efficiency and selectivity for a variety of thiophene substrates [36,62]. Under such conditions, we boosted the yield from 55% (entry 7) to 68% (entry 10) when working with Na_2_CO_3_ as the base. The use of *t*BuONa further improved the yield to a remarkable 80% (entry 12). As for the cases of the reactions performed with pivalic acid, the use of NaOH instead of *t*BuONa (entry 11) led to lower yields (77%). According to recent reports, the use of a large excess of base is beneficial; we thus doubled the amount of *t*BuONa (entry 13) [52]. Indeed, the yield was further improved to 88%. Having assessed the relevant role of the partition of the carboxylic additive, we wondered whether the presence of the phase transfer agent was still necessary. Its complete removal (Entry 14) proved to be detrimental, as the yield dropped to 59%, mostly due to limited conversion.

**Scheme 3:**
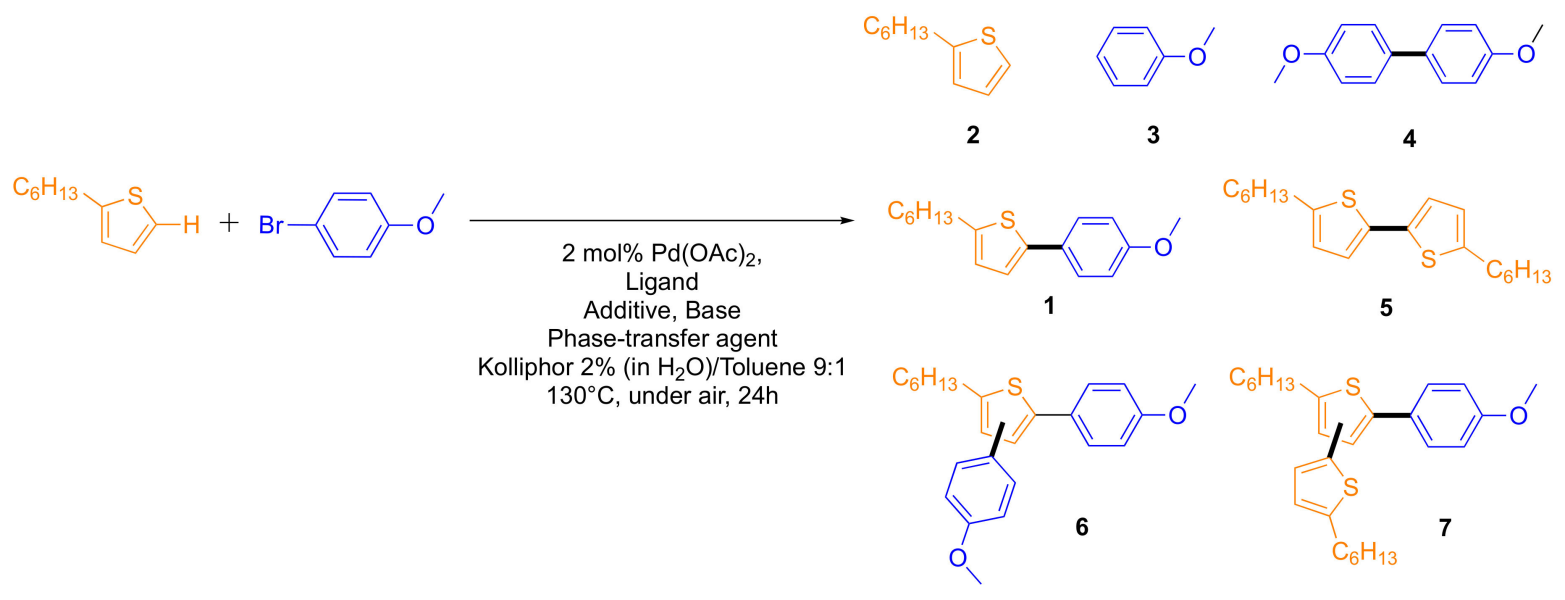
Product distribution in the DHA model reaction.

Having identified viable conditions with which to carry out the reaction, we then turned to the identification of the byproducts and to a further fine tuning of conditions aimed at suppressing the most detrimental processes in view of a possible extension of the method to polymerizations: dehalogentation (formation of anisole **3**), homocoupling of the bromide (4,4′-dimethoxybiphenyl **4**), oxidative homocoupling of the aryl (5,5′-dihexyl-2,2′-bithiophene **5**), double arylation of thiophene (derivatives **6**, mixture of regioisomers) and oxidative coupling of product **4** and 2-hexylthiophene (derivatives **7**, mixture of regioisomers); see Scheme 3 and Table 2.

Having identified viable conditions with which to carry out the reaction, we then turned to the identification of the byproducts and to a further fine tuning of conditions aimed at suppressing the most detrimental processes in view of a possible extension of the method to polymerizations: dehalogentation (formation of anisole **3**), homocoupling of the bromide (4,4′-dimethoxybiphenyl **4**), oxidative homocoupling of the aryl (5,5′-dihexyl-2,2′-bithiophene **5**), double arylation of thiophene (derivatives **6**, mixture of regioisomers) and oxidative coupling of product **4** and 2-hexylthiophene (derivatives **7**, mixture of regioisomers); see Scheme 3 and Table 2.

Entry 1 of Table 2 shows that under the best experimental conditions we identified, the most relevant impurities are the unreacted 2-hexylthiophene (3%) and the double arylation derivative (4%). The increase in the amounts of carboxylic additive and phase transfer agent did not influence the product distribution in a sizeable way (entry 2); as such we maintained the usual 0.3 eq stoichiometry for the remaining attempts. The substitutions of Cy_3_PHBF_4_ with tri-*tert*-butylphosphine tetrafluoroborate salt (*t*Bu_3_PHBF_4_), another popular sterically-hindered phosphine employed in DHA, led to a slightly lower conversion without affecting the product distribution (entry 3). Given the fact that NDA potassium salt is also a surfactant, in entry 4 we decided to remove the K-EL. The reaction performed very similarly to entry 1, with a measurable increase in the formation of the dithiophenes **5** and **7**. Both derivatives are formed according to an oxidative coupling of thiophene derivatives, requiring the presence of a Pd^2+^ species. According to our previous experience with K-EL in S–M and B–H couplings, the main advantage of using such a surfactant is precisely the suppression of oxidative side reactions even under an oxygenated environment [42,63]. In order to prove the point, we repeated the K-EL free reaction under nitrogen atmosphere and with nitrogen saturated solvents (entry 5), correctly observing essentially the same product distributions of entry 1 reaction. Even if in absolute terms the incidence of all byproducts is minimal, the influence of their formation in a polymerization reaction would be sizeable, as the pathway leading to derivative **3** is a termination reaction. those leading to **4** and **5** would introduce defects and those leading to **6** and **7**, crosslinking.

### 2.2. Scope and Generality of the Method in the Preparation of Conjugated Building Blocks

**Scheme 4:**
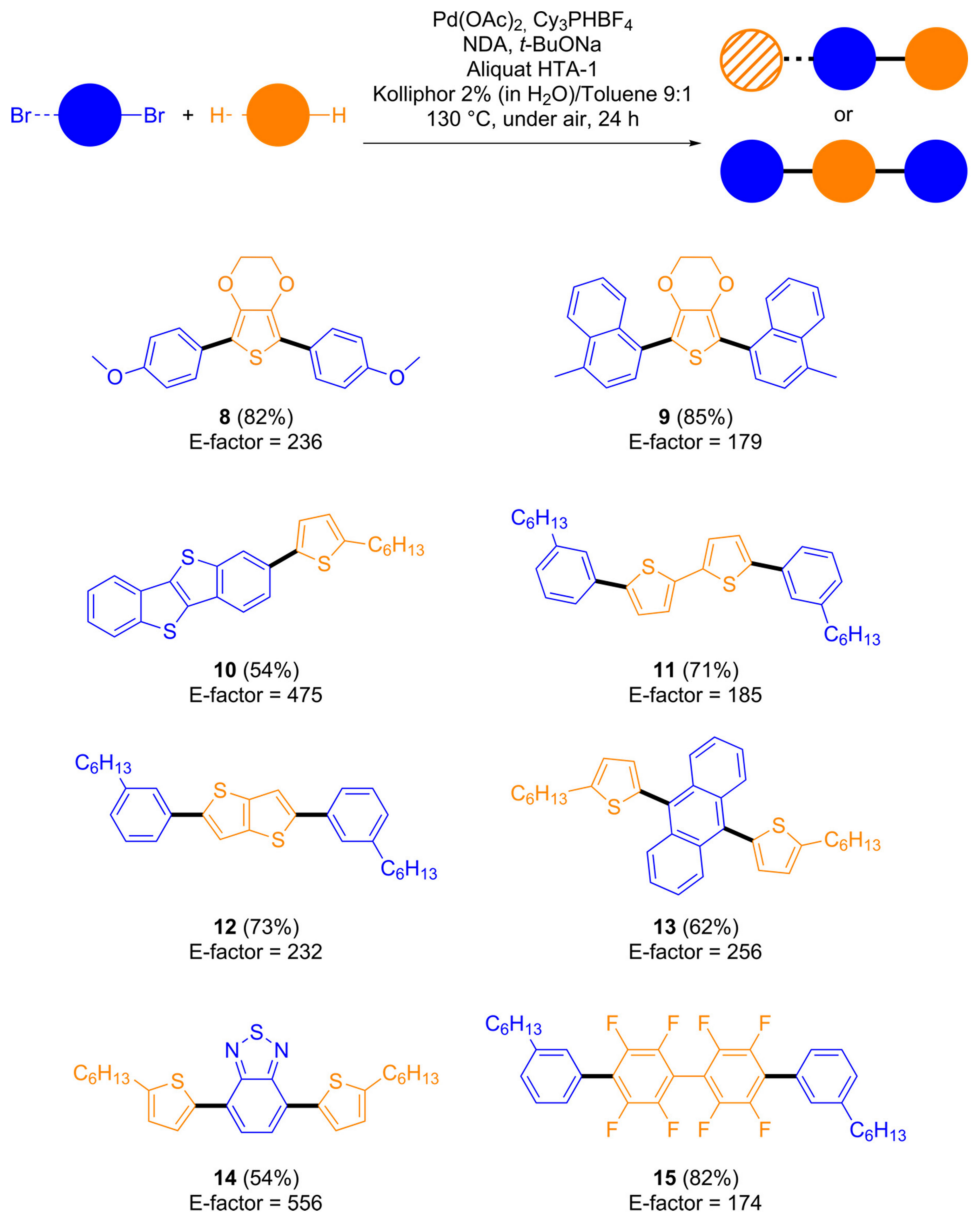
Chemical structures of π-conjugated building blocks synthetized by DHA with the emulsion optimized conditions.

Having optimized the method on the model, thiophene containing derivative **1**, we selected a series of representative molecular derivatives possessing different electronic characteristics. The general structure of organic semiconductors features the alternation of aromatic and heteroaromatic rings having accepting, donating and borderline characteristics due to the presence of electron donating or electron accepting groups or the intrinsic characteristics of the various heteroaromatics. Such characteristics have an effect on the selectivity and efficiency of the DHA reaction; we thus explored the synthesis of eight compounds featuring the most representative motifs encountered in biaryls for organic semiconductors (Scheme 4). Table 3 summarizes the most relevant optical and electrochemical features of all compounds (LUMO levels are not reported as no reversible reduction peak was observed for any compound except derivative **13**). Absorption, steady state emission and differential pulsed voltammetry, alongside with details on the syntheses for all compounds are included in the Supplementary Materials Section.

Derivatives **8** and **9** feature a low oxidation potential, having HOMO levels of −5.18 and −5.49 eV and are representatives of p-type organic semiconductors. They were prepared by reacting 3,4-ethylenedioxythiophene (EDOT) with the electron rich bromide 4-bromoanisole and the sterically hindered 1-bromo-4-methylnaphthalene. As already reported in the literature, EDOT turned out to be an excellent target for direct arylation and we obtained **8** and **9** in 82% and 85% yields. Derivative **10** is a known p-type semiconductor featuring the popular [1]benzothieno[3,2-b][1]benzothiophene core (BTBT). It is a particularly noteworthy synthetic target, as its synthesis and the corresponding E-factors were already reported in the literature by Stille coupling in organic solvent (yield 77%, E-factor 1137) [64] and by Suzuki–Miyaura micellar (yield 91%, E-factor 39) and emulsion (yield 80%, E-factor 231) techniques, thereby enabling a direct evaluation of the advantages of the emulsion DHA protocol [46]. Under DHA emulsion conditions, we isolated **10** in 54% yield by the reaction of 2-bromo-BTBT with 2-hexylthiophene, with an E-factor of 475 after chromatographic purification. The comparison seems to be unfavorable for DHA, yet it has to be stressed that in this case no activation of the thiophene ring is required. Just to give an example, the preparation of 5-hexyl-2-thiopheneboronic acid pinacol ester from 2-hexyl-5-bromothiophene alone has an E-Factor in the order of 100 [65].

Derivatives **11**–**13** also have a potential p-type character, as assessed by the corresponding HOMO levels of −5.44, −5.54 and −5.59. Derivatives **11** and **12**, featuring a dithienyl and a dithieno bridge, showcase the robustness of the approach towards more extended conjugated bridges. As the aryl bromide, we selected 3-bromohexylbenzene in order to decouple the electron donating effect of the alkyl solubilizing chain with respect to the reactive halogenated site. Yields were similar with derivative **12** (73%) slightly outperforming **11** (71%). Derivative **13** is a representative of the well-known 9,10-diarylanthracene fluorescent probes. It was prepared in 62% yield by reacting 2-hexylthiophene with 9,10-dibromoanthracene, demonstrating the capability to perform the DHA coupling twice even on severely sterically hindered sites. Finally, derivatives **14** and **15** are examples of donor–acceptor compounds featuring the alternation of electron rich and electron poor rings. We prepared **14** in 54% yield by reacting 2-hexylthiophene with 2,1,3-benzothiadiazole, an heteroaromatic frequently employed in the design of low band gap polymers. Finally, we prepared the fluorinated derivative **15** by the reaction of 3-bromohexylbenzene with 2,2′,3,3′,5,5′,6,6′-octafluorobiphenyl in 82% yield, according to the known high reactivity of heavily fluorinated aryls in DHA [58].

## 3. Materials and Methods

All reagents, chemical compounds and solvents were purchased from TCI Europe (Haven, Belgium), Fluorochem Europe (Hadfield, Derbyshire, United Kingdom), Alfa Aesar Europe (Kandel, Germany) and Merck Life Science S.r.l. (Milano, Italy) and used as received without any further purification. In particular, for the catalysts, Pd(OAc)_2_ was purchased from Fluorochem, Cy_3_PHBF_4_ was purchased from Alfa Aesar Europe (Kandel, Germany) and *t*Bu_3_PHBF_4_ was purchased from Fluorochem Europe (Hadfield, Derbyshire, United Kingdom).

Unless otherwise stated, all reactions were carried out in a pressure-tight 10 mL screw-cap glass tube equipped with a cylindrical stirring bar in vertical position, under magnetic stirring at 1000 rpm for 24 h and at a nominal concentration for the reagents of 0.5 mol/L. Reactions described in Table 1 and Table 2 were extracted with CH_2_Cl_2_, filtered on a pad of silica gel and submitted to GC–MS characterization. Yields of product and byproducts were assessed by GC–MS using a simple semiquantitative area normalization method.

Chromatographic purifications were performed using Davisil LC 60A silica gel (pore size 60 Å, 70−200 μm). Compositions of solvent mixtures used as eluents are indicated as volume/volume ratios. Melting points were determined using a Buchi M-560 (BUCHI Italia s.r.l, Cornaredo, Italy) apparatus and are uncorrected. GC–MS spectra were collected on a Clarus 560 S PerkinElmer (Perkin Elmer Italia, Milano, Italy) having an Elite-5MS 30.0 m × 250 μm column. Helium was used as carrier gas. ^1^H-NMR and ^13^C-NMR spectra were collected on a Bruker NMR Avance 400 NEO spectrometer; coupling constants are reported in Hz. Absorption spectra of derivatives **1** and **8**–**15** were collected on a Cary 60 UV−Vis Agilent spectrophotometer in a 10 mm path length quartz cuvette. Photoluminescence (PL) spectra of derivatives **1**, **8**–**15** were collected on a Cary Eclipse Fluorescence Agilent spectrophotometer (Santa Clara, Califoria, United States) in a 10 mm path length quartz cuvette. Differential pulsed voltammetry (DPV) characterizations were performed in glove box in a three-electrode glass cell using a glassy carbon pin as the working electrode (WE, diameter of 2 mm), a platinum mesh as the counter electrode (CE) and Ag/AgCl wire as the quasi-reference electrode (QRE), using an EG&G Princeton Applied 2273 potentiostat/galvanostat. The electrolyte solution ((Bu_4_N)ClO_4_ 0.1 M in CH_2_Cl_2_, referred to as TCDCM01), sample solutions (0.005 M in TCDCM01) and the calibration solution (Ferrocene 0.001 M in TCDCM01), were prepared and stored in the glove box. HOMO values were calculated from the half-wave maximum of the DPV plot. The potential values of −4.6 eV for NHE vs. vacuum and of 0.2 V for Fc/Fc^+^ vs. NHE were used in potential/energy conversions [66].

## 4. Conclusions

We have shown that the 9:1 *v*/*v* emulsion of 2 wt% K-EL solution in water and toluene is an ideal medium to carry out DHA reactions on thiophene derivatives. In particular, we have demonstrated that while moving from the common homogeneous phase reactions in high-boiling-point organic solvents, the colocalization of reagents, catalyst and additives becomes the key factor in achieving high conversions. To the best of our knowledge, we employed NDA for the first time instead of pivalic acid precisely to control repartition of reactive species between the water and oil phase constituting the reaction mixture while working under emulsion conditions. The amount of toluene required to successfully carry out the reaction is so low that said organic solvent can be considered less than stoichiometric with respect to the aryls involved. Once optimized for the model reaction between 4-bromoanisole and 2-hexylthiophene, the method was successfully extended to the preparation of eight model molecular semiconductors possessing structural features representative of common organic semiconductors for plastic (opto)electronics. We characterized all such compounds in terms of optical and electrochemical properties, and we calculated the E-factor for their preparation. We obtained values in the 10^2^ order, which is compatible with the requirements for scaling up.

## Figures and Tables

**Table 1:** Screening of suitable reaction conditions and formulation of the DHA coupling between 2-hexylthiophene and 4-bromoanisole in emulsion medium. All reactions were carried out in a pressure-tight 10 mL screw-cap glass tube under magnetic stirring at 1000 rpm for 24 h and at a nominal concentration for the reagents of 0.5 mol/L.

Entry	Additive (30 mol%)	Base 1.5 eq	Phase-Transfer Agent (30 mol%)	T (°C)	Conversion to Product (%)
1	PivOH	Na_2_CO_3_	_	80	trace
2 ^a^	PivOK	_	_	80	trace
3 ^a^	PivONBu_4_	_	_	80	trace
4	PivOH	Na_2_CO_3_	_	130	30
5	PivOH	Cs_2_CO_3_	_	130	27
6	PivOH	Na_2_CO_3_	Aliquat 336	130	53 (44 isolated)
7	PivOH	Na_2_CO_3_	Aliquat HTA-1	130	55
8	PivOH	NaOH	Aliquat HTA-1	130	48
9	PivOH	*t*BuONa	Aliquat HTA-1	130	59
10	NDA	Na_2_CO_3_	Aliquat HTA-1	130	68
11	NDA	NaOH	Aliquat HTA-1	130	77
12	NDA	*t*BuONa	Aliquat HTA-1	130	80
13 ^b^	NDA	*t*BuONa	Aliquat HTA-1	130	88 (86 isolated)
14 ^b^	NDA	*t*BuONa	-	130	59

^a^ One equivalent amount of pivalate salt employed; ^b^ 3 equiv. of *t*BuONa employed.

**Table 2:** Optimization of reaction conditions for the DHA coupling between 2-hexylthiophene and 4-bromoanisole in the emulsion medium. All reactions were carried out in a pressure-tight 10 mL screw-cap glass tube under magnetic stirring at 1000 rpm for 24 h and at a nominal concentration for the reagents of 0.5 mol/L. RCOOH stands for the carboxylic additive.

Entry	Ligand (4 mol%)	RCOOH (eq)	Base (eq)	Phase-Transfer Agent (eq)	1	2	3	4	5	6	7
1	Cy_3_PHBF_4_	NDA	*t*BuONa	Aliquat HTA-1	88%	3%	2%	1%	2%	4%	0%
		0.3	3	0.3							
2	Cy_3_PHBF_4_	NDA	*t*BuONa	Aliquat HTA-1	87%	3%	1%	2%	2%	3%	1%
		1	3	1							
3	*t*Bu_3_PHBF_4_	NDA	*t*BuONa	Aliquat HTA-1	83%	9%	3%	1%	2%	2%	0%
		0.3	3	0.3							
4 ^a^	Cy_3_PHBF_4_	NDA	*t*BuONa	Aliquat HTA-1	85%	3%	1%	1%	4%	4%	3%
		0.3	3	0.3							
5 ^a,b^	Cy_3_PHBF_4_	NDA	*t*BuONa	Aliquat HTA-1	86%	4%	2%	1%	2%	4%	1%
		0.3	3	0.3							

^a^ Reaction performed without K-EL; ^b^ reaction performed under nitrogen atmosphere.

**Table 3:** Optical and electrochemical properties of compounds **1**, **8**–**15** in CH_2_Cl_2_: linear absorption maxima (λ_max_(abs)), steady state emission maxima(λ_max_(em), half-wave potentials (E_1/2_ vs. Fc/Fc^+^, supporting electrolyte tetrabutylammonium perchlorate 0.1 M) and electrochemical HOMO levels (eV).

Derivative	λ_max_ (abs) [nm]	λ_max_ (em) [nm]	Stokes Shift [eV]	E_1/2_ (V)	HOMO (eV)
**8**	351	392	0.37	0.38	−5.18
**9**	334	430	0.83	0.69	−5.49
**10**	364	379	0.14	0.78	−5.58
**11**	375	430	0.43	0.64	−5.44
**12**	351	391	0.36	0.74	−5.54
**13**	401	461	0.40	0.78	−5.59
**14**	469	603	0.58	−1.78	−3.00
**15 ^a^**	267	341	1.00	-	-

^a^ The compound has no redox processes within the stability window of the electrolyte.

## Supplementary Materials

The supplementary materials are available online. Details on the synthesis of derivatives **1**, **8**–**15**. E-factor calculations. UV–Vis absorption and PL spectra of derivatives **1** and **8**–**15**. DPV plots of derivatives **8**–**15**. ^1^H-NMR and ^13^C-NMR spectra of derivatives **1** and **8**–**15**.
