# Supplementary material for: Sustainable Access to π-Conjugated Molecular Materials via Direct (Hetero)Arylation Reactions in Water and under Air
Source: Molecules. 2020 Aug 14;25(16):3717. doi: 10.3390/molecules25163717 (PMC7465621; doi:10.3390/molecules25163717)
Supplement: Supplementary file 1 [file molecules-25-03717-s001.pdf]

Supporting Information:

Sustainable access to π-Conjugated Molecular Materials via Direct (Hetero)Arylation Reactions in Water and under Air

Adiel Mauro Calascibetta,[1] Sara Mattiello,[2] Alessandro Sanzone,[1] Irene Facchinetti,[1] Mauro Sassi,[2] and Luca Beverina[2,*]

Department of Materials Science, University of Milano-Bicocca, Via R. Cozzi, 55, Milano I-20125, Italy

Department of Materials Science, University of Milano-Bicocca and INSTM. Via R. Cozzi, 55, Milano I-20125, Italy

* Correspondence: luca.beverina@unimib.it

Contents

1 General procedure for emulsion couplings, details on single reactions, and E-factor calculations……………………………………………………...…..S-1

2 UV-Vis absorption and PL spectra for derivatives 1, 8-15……….………S-7

3 DPV plots of derivatives 8-15……………………………..……..………S-16

4 ^{1}H and ^{13}C NMR spectra for derivatives 1, 8-15……………………..…..S-24

1 General procedure for emulsion couplings, details on single reactions, and E-factor calculations

1 L of K-EL 2 wt%:toluene (9:1 v/v) emulsion is prepared homogenizing a 2 wt% aqueous dispersion of K-EL (18 g of Kolliphor EL in 882 mL of deionized water) with 100 mL of toluene using a T 25 digital ULTRA-TURRAX® until a stable, milky dispersion is obtained. All reactions were carried out in a pressure-tight 10 mL screw-cap glass tube under magnetic stirring at 1000 rpm for 24 hrs and at a nominal concentration for the limiting reagent of 0.5 mol/L.

As the reported reactions are palladium mediated C-C couplings, water has always been taken into account for the calculation of the E-factor. In fact, it can be contaminated with palladium, and it therefore must be considered an hazardous waste. For this reason, inorganic bases used in the reactions are included as well in the waste calculation, as they remain dissolved in the aqueous phase.

2.1 2-(4-methoxyphenyl)-5-hexylthiophene (model compound, 1)

2-hexylthiophene (0.168 g, 1.0 mmol), 4-bromoanisole (0.187 g, 1.0 mmol), $Pd(OAc)_2$ (0.005 g, 0.02 mmol), Cy_3PHBF_4 (0.015 g, 0.04 mmol), *t*BuONa (0.288 g, 3.0 mmol), neodecanoic acid (0.052 g, 0.3 mmol), Aliquat HTA-1 35%wt in H_2O (0.226 g, 0.3 mmol) are weighted in the pressure-tight 10 mL screw-cap glass tube, then 2 mL of K-EL 2 wt%-toluene (9:1 v/v, 2 mL) oil-in-water emulsion are added. The reaction mixture is stirred and allowed to homogenize for 5 min at RT, subsequently it is heated under stirring at 130°C through oil bath for 24 h. The mixture is cooled, then diluted with 8 mL of water and filtered. The crude was purified by column chromatography (17.75 g of silica) using a mixture of petroleum ether/ dichloromethane 7:3 (71 mL) as eluent. The pure product was isolated as a brownish solid in 84% yield (231 mg, 0.84 mmol). Mp: 58-59 °C. ^1H NMR (400 MHz, CDCl3): δ 7.52 (d, J=8.4 Hz, 2H), 7.03 (d, J=3.6 Hz, 1H), 6.92 (d, J=8.4 Hz, 2H), 6.74 (d, J=3.6 Hz, 1H), 3.84 (s, 3H), 2.84 (t, J=7.5 Hz, 2H), 1.77–1.70 (m, 2H), 1.45–1.32 (m, 6H), 0.95 (t, J=7.5 Hz, 3H). ^{13}C NMR (100 MHz, $CDCl_3$): δ 158.8, 144.7, 141.5, 127.7, 127.5, 127.3, 126.9, 126.7, 124.8, 121.6, 114.5, 114.2, 113.8, 55.3, 31.63, 31.59, 30.2, 28.8, 22.6, 14.1.

<u>E-factor calculation</u>

$$E_{factor} = \frac{mass\ of\ reagents + mass\ of\ auxiliaries - mass\ of\ product}{mass\ of\ product} =$$

$$= \frac{2.9144 + 86.881 - 0.231}{0.231} = 388$$

2.2 2,3-dihydro-5,7-bis(4-methoxyphenyl)thieno[3,4-b][1,4]dioxine (8)

3,4-ethylenedioxythiophene (0.142 g, 1.0 mmol), 4-bromoanisole (0.374 g, 2.0 mmol), $Pd(OAc)_2$ (0.009 g, 0.04 mmol), Cy_3PHBF_4 (0.030 g, 0.08 mmol), *t*BuONa (0.288 g, 3.0 mmol), neodecanoic acid (0.104 g, 0.6 mmol), Aliquat HTA-1 35%wt in H_2O (0.452 g, 0.6 mmol) are weighted in the pressure-tight 10 mL screw-cap glass tube, then 2 mL of K-EL 2 wt%-toluene (9:1 v/v, 2 mL) oil-in-water emulsion are added. The reaction mixture is stirred and allowed to homogenize for 5 min at RT, subsequently it is heated under stirring at 130°C through oil bath for 24 h. The mixture is cooled, then diluted with 8 mL of methanol and filtered. The crude was purified by filtration on a pad of silica gel (15 g) with CH_2Cl_2 (30 mL). The pure product was obtained by recrystallization from toluene (5 mL) and isolated as a yellowish solid in 82% yield (291 mg, 0.82 mmol). Mp: 178-179 °C. ^{1}H NMR (400 MHz, CD_2Cl_2): δ 7.70 (d, J = 8.82 Hz, 4H), 6.95 (d, J = 8.82 Hz, 4H), 4.37 (s, 4H), 3.86 (s, 6H). ^{13}C NMR (100 MHz, CD_2Cl_2): δ 158.4, 137.8, 127.2, 125.8, 114.0, 113.8, 64.6, 55.3.

<u>E-factor calculation</u>

$$E_{factor} = \frac{mass\ of\ reagents + mass\ of\ auxiliaries - mass\ of\ product}{mass\ of\ product} =$$

$$= \frac{3.3724 + 65.571 - 0.291}{0.291} = 236$$

2.3 2,3-dihydro-5,7-bis(4-methylnaphthyl)thieno[3,4-b][1,4]dioxine (9)

3,4-ethylenedioxythiophene (0.142 g, 1.0 mmol), 1-bromo-4-methylnaphtalene (0.442 g, 2.0 mmol), $Pd(OAc)_2$ (0.009 g, 0.04 mmol), Cy_3PHBF_4 (0.030 g, 0.08 mmol), *t*BuONa (0.288 g, 3.0 mmol), neodecanoic acid (0.104 g, 0.6 mmol), Aliquat HTA-1 35%wt in H_2O (0.452 g, 0.6 mmol) are weighted in the pressure-tight 10 mL screw-cap glass tube, then 2 mL of K-EL 2 wt%-toluene (9:1 v/v, 2 mL) oil-in-water emulsion are added. The reaction mixture is stirred and allowed to homogenize for 5 min at RT, subsequently it is heated under stirring at 130°C through oil bath for 24 h. The mixture is cooled, then diluted with 8 mL of methanol and filtered. The crude was purified by filtration on a pad of silica gel (15 g) with CH_2Cl_2 (30 mL). The pure product was isolated as a pale-yellow solid in 85% yield (360 mg, 0.85 mmol). Mp: 240-241 °C. ^{1}H NMR (400 MHz, $CDCl_3$) δ 8.26 – 8.24 (m, 2H), 8.10 – 8.07 (m, 2H), 7.61 – 7.58 (m, 6 H), 7.41 (d, *J* = 7.2 Hz, 2H), 4.27 (s, 4H), 2.76 (s, 6H). ^{13}C NMR (100 MHz, $CDCl_3$) δ 137.72, 135.05, 132.95, 131.90, 128.80, 128.09, 127.13, 126.28, 125.88, 125.87, 124.47, 114.79, 64.71, 19.65. Anal. Calcd for $C_{28}H_{22}O_2S$: C, 79.59; H, 5.25; O, 7.57. Found: C, 79.73; H, 5.43; O, 7.28.

E-factor calculation

$$E_{factor} = \frac{mass\ of\ reagents + mass\ of\ auxiliaries - mass\ of\ product}{mass\ of\ product} =$$
$$= \frac{3.4404 + 61.236 - 0.360}{0.360} = 179$$

2.4 2-(5-hexyl)-2-thienyl-[1]benzothieno[3,2-b][1]benzothiophene (10)

2-bromo-[1]benzothieno[3,2-b][1]benzothiophene (0.320 g, 1.0 mmol), 2-hexylthiophene (0.168 g, 1.0 mmol), $Pd(OAc)_2$ (0.005 g, 0.02 mmol), Cy_3PHBF_4 (0.015 g, 0.04 mmol), *t*BuONa (0.288 g, 3.0 mmol), neodecanoic acid (0.052 g, 0.3 mmol), Aliquat HTA-1 35%wt in H_2O (0.226 g, 0.3 mmol) are weighted in the pressure-tight 10 mL screw-cap glass tube, then 2 mL of K-EL 2 wt%-toluene (9:1 v/v, 2 mL) oil-in-water emulsion are added. The reaction mixture is stirred and allowed to homogenize for 5 min at RT, subsequently it is heated under stirring at 130°C through oil bath for 24 h. The mixture is cooled, then diluted with 8 mL of methanol and filtered. The crude was purified by column chromatography (24.4 g of silica) using a mixture of petroleum ether/dichloromethane 9:1 (97.5 mL) as eluent. The pure product was isolated as a pale-yellow solid in 54% yield (0.220 g, 0.54 mmol). Mp: 184-185 °C (degradation). ^{1}H NMR (400 MHz, $CDCl_3$) δ 8.08 (d, *J* = 1.3 Hz, 1H), 7.92 (d, *J* = 8.0 Hz, 1H), 7.88 (d, *J* = 7.7 Hz, 1H), 7.84 (d, *J* = 8.3 Hz, 1H), 7.66 (dd, *J* = 8.3, 1.6 Hz, 1H), 7.48 – 7.44 (m, 1H), 7.43 – 7.38 (m, 1H), 7.22 (d, *J* = 3.5 Hz, 1H), 6.79 (d, *J* = 3.6 Hz, 1H), 2.85 (t, *J* = 7.6 Hz, 2H), 1.73 (dt, *J* = 15.3, 7.6 Hz, 2H), 1.46 – 1.29 (m, 6H), 0.91 (t, *J* = 7.1 Hz, 3H). ^{13}C NMR (126 MHz, $CDCl_3$)* δ 147.10, 143.97, 143.13, 142.04, 134.37, 134.20, 133.99, 132.89, 132.72, 126.11, 125.85, 125.80, 124.90, 123.98, 123.82, 122.61, 122.41, 121.26, 32.50, 32.47, 31.19, 29.67, 23.47, 14.97.

E-factor calculation

$$E_{factor} = \frac{mass\ of\ reagents + mass\ of\ auxiliaries - mass\ of\ product}{mass\ of\ product} =$$
$$= \frac{3.0474 + 101.691 - 0.220}{0.220} = 475$$

2.5 5,5'-bis(3-hexylphenyl)-2,2'-bithiophene (11)

2,2’-bithiophene (0.166 g, 1.0 mmol), 1-bromo-3-hexylbenzene (0.482 g, 2.0 mmol), $Pd(OAc)_2$ (0.009 g, 0.04 mmol), Cy_3PHBF_4 (0.030 g, 0.08 mmol), *t*BuONa (0.288 g, 3.0 mmol), neodecanoic acid (0.104 g, 0.6 mmol), Aliquat HTA-1 35%wt in H_2O (0.452 g, 0.6 mmol) are weighted in the pressure-tight 10 mL screw-cap glass tube, then 2 mL of K-EL 2 wt%-toluene (9:1 v/v, 2 mL) oil-in-water emulsion are added. The reaction mixture is

* In this case only, the spectrum was acquired with a Bruker NMR 500 Avance.

stirred and allowed to homogenize for 5 min at RT, subsequently it is heated under stirring at 130°C through oil bath for 24 h. The mixture is cooled, then diluted with 8 mL of methanol and filtered. The crude was purified by filtration on a pad of silica gel (15 g) with CH_2Cl_2 (30 mL). The pure product was isolated as a yellowish solid in 71% yield (348 mg, 0.71 mmol). Mp: 111-112 °C. ^{1}H NMR (400 MHz, $CDCl_3$) δ 7.45 – 7.41 (m, 4H), 7.32 – 7.27 (m, 2H), 7.24 (d, *J* = 3.8 Hz, 2H), 7.17 (d, *J* = 3.8 Hz, 2H), 7.12 (dt, *J* = 7.6, 1.2 Hz, 2H), 2.65 (t, *J* = 7.6 Hz, 4H), 1.66 (m, 4H), 1.44 – 1.27 (m, 12H), 0.90 (t, *J* = 7.1 Hz, 6H). ^{13}C NMR (100 MHz, $CDCl_3$) δ 143.72, 143.37, 136.58, 133.93, 128.82, 127.80, 125.71, 124.32, 123.65, 122.98, 35.97, 31.73, 31.44, 29.02, 22.61, 14.10. Anal. Calcd for $C_{32}H_{38}S_2$: C,78.96; H, 7.87. Found: C, 78.60; H, 7.91.

<u>E-factor calculation</u>

$$E_{factor} = \frac{mass\ of\ reagents + mass\ of\ auxiliaries - mass\ of\ product}{mass\ of\ product} =$$
$$= \frac{3.5044 + 61.236 - 0.348}{0.348} = 475$$

2.6 2,5-bis(3-hexylphenyl)thieno[3,2-b]thiophene (12)

Thieno[3,2-b]thiophene (0.140 g, 1.0 mmol), 1-bromo-3-hexylbenzene (0.482 g, 2.0 mmol), $Pd(OAc)_2$ (0.009 g, 0.04 mmol), Cy_3PHBF_4 (0.030 g, 0.08 mmol), *t*BuONa (0.288 g, 3.0 mmol), neodecanoic acid (0.104 g, 0.6 mmol), Aliquat HTA-1 35%wt in H_2O (0.452 g, 0.6 mmol) are weighted in the pressure-tight 10 mL screw-cap glass tube, then 2 mL of K-EL 2 wt%-toluene (9:1 v/v, 2 mL) oil-in-water emulsion are added. The reaction mixture is stirred and allowed to homogenize for 5 min at RT, subsequently it is heated under stirring at 130°C through oil bath for 24 h. The mixture is cooled, then diluted with 8 mL of methanol and filtered. The crude was purified by filtration on a pad of silica gel (15 g) with CH_2Cl_2 (30 mL). The product was recrystallized from heptane (20 mL) and isolated as a golden solid in 73% yield (336 mg, 0.73 mmol). Mp: 130-131 °C. ^{1}H NMR (400 MHz, $CDCl_3$) δ 7.48 – 7.44 (m, 6H), 7.34 – 7.28 (m, 2H), 7.15 – 7.11 (m, 2H), 2.66 (t, *J* = 7.6 Hz, 4H), 1.71-1.62 (m, 4H), 1.40-1.31 (m, 12H), 0.91 (t, *J* = 7.1 Hz, 6H). ^{13}C NMR (100 MHz, $CDCl_3$) δ 146.01, 143.76, 139.14, 134.62, 128.87, 127.96, 125.85, 123.17, 115.38, 35.97, 31.72, 31.42, 29.02, 22.61, 14.09. Anal. Calcd for $C_{30}H_{36}S_2$: C,78.21; H, 7.88. Found: C, 78.03; H, 7.99.

<u>E-factor calculation</u>

$$E_{factor} = \frac{mass\ of\ reagents + mass\ of\ auxiliaries - mass\ of\ product}{mass\ of\ product} =$$
$$= \frac{3.4784 + 74.916 - 0.336}{0.336} = 232$$

2.7 9,10-bis(5-hexyl-2-thienyl)anthracene (13)

9,10-dibromoanthracene (0.336 g, 1.0 mmol), 2-hexylthiophene (0.337 g, 2.0 mmol), $Pd(OAc)_2$ (0.009 g, 0.04 mmol), Cy_3PHBF_4 (0.030 g, 0.08 mmol), *t*BuONa (0.288 g, 3.0 mmol), neodecanoic acid (0.104 mg, 0.6 mmol), Aliquat HTA-1 35%wt in H_2O (0.452 g, 0.6 mmol) are weighted in the pressure-tight 10 mL screw-cap glass tube, then 2 mL of K-EL 2 wt%-toluene (9:1 v/v, 2 mL) oil-in-water emulsion are added. The reaction mixture is stirred and allowed to homogenize for 5 min at RT, subsequently it is heated under stirring at 130°C through oil bath for 24 h. The mixture is cooled, then diluted with 8 mL of methanol and filtered. The crude was purified by filtration on a pad of silica gel (15 g) with CH_2Cl_2 (30 mL). The product was recrystallized from heptane (24 mL) and isolated as a yellowish solid in 62% yield (316 mg, 0.73 mmol). Mp: 114-115 °C. ^1H NMR (400 MHz, $CDCl_3$) δ 8.01 – 7.88 (m, 4H), 7.46 – 7.36 (m, 4H), 7.00 (d, J = 3.3 Hz, 2H), 6.97 (dt, J = 3.4, 0.9 Hz, 2H), 2.96 (t, , *J* = 7.6 Hz, 4H), 1.89 – 1.74 (m, 4H), 1.58 – 1.32 (m, 12H), 0.94 (t, J = 7.1 Hz, 6H). ^{13}C NMR (100 MHz, $CDCl_3$) δ 147.44, 136.11, 131.47, 130.65, 129.16, 126.80, 125.47, 123.92, 31.69, 31.61, 30.28, 28.93, 22.61, 14.09.

<u>E-factor calculation</u>

$$E_{factor} = \frac{mass\ of\ reagents + mass\ of\ auxiliaries - mass\ of\ product}{mass\ of\ product} =$$

$$= \frac{3.5284 + 77.652 - 0.316}{0.316} = 256$$

2.8 4,7-bis(5-hexyl-2-thienyl)benzo[c][1,2,5]thiadiazole (14)

4,7-dibromo-benzo[1,2,5]thiadiazole (0.294 g, 1.0 mmol), 2-hexylthiophene (0.337 g, 2.0 mmol), $Pd(OAc)_2$ (0.009 g, 0.04 mmol), Cy_3PHBF_4 (0.030 g, 0.08 mmol), *t*BuONa (0.288 g, 3.0 mmol), neodecanoic acid (0.104 mg, 0.6 mmol), Aliquat HTA-1 35%wt in H_2O (0.452 g, 0.6 mmol) are weighted in the pressure-tight 10 mL screw-cap glass tube, then 2 mL of K-EL 2 wt%-toluene (9:1 v/v, 2 mL) oil-in-water emulsion are added. The reaction mixture is stirred and allowed to homogenize for 5 min at RT, subsequently it is heated under stirring at 130°C through oil bath for 24 h. The mixture is cooled, then diluted with 8 mL of methanol and filtered. The crude was purified by column chromatography (31.5 g of silica) using a mixture of petroleum ether/dichloromethane 8:2 (126 mL) as eluent. Mp: 75-76 °C. The pure product was isolated as a red solid in 54% yield (254 mg, 0.54 mmol). ^1H NMR (400 MHz, $CDCl_3$) δ 7.89 (d, *J* = 3.7 Hz, 1H), 7.67 (s, 1H), 6.84 (dt, *J* = 3.7, 0.9 Hz, 1H), 2.87 (t, *J* = 7.5 Hz, 2H), 1.89 – 1.59 (m, 2H), 1.48 – 1.32 (m, 6H), 0.93 (t, *J* = 7.1 Hz, 3H). ^{13}C NMR (100 MHz, $CDCl_3$) δ 152.53, 147.60, 136.85, 127.32, 125.61, 125.14, 124.99, 31.64, 31.60, 30.32, 28.90, 22.64, 14.14. Anal. Calcd for $C_{28}H_{34}S_3$: C, 72.05; H, 7.34. Found: C, 72.21; H, 7.65.

E-factor calculation

$$E_{factor} = \frac{mass\ of\ reagents + mass\ of\ auxiliaries - mass\ of\ product}{mass\ of\ product} =$$
$$= \frac{3.0474 + 137.88 - 0.254}{0.254} = 556$$

2.9 1,4′-bis(3-hexylphenyl)-2′,2″,3′,3″,5′,5″,6′,6″-octafluorobiphenyl (15)

2,2′,3,3′,5,5′,6,6′-octafluorobiphenyl (0.298 g, 1.0 mmol), 1-bromo-3-hexylbenzene (0.482 g, 2.0 mmol), $Pd(OAc)_2$ (0.009 g, 0.04 mmol), Cy_3PHBF_4 (0.030 g, 0.08 mmol), *t*BuONa (0.288 g, 3.0 mmol), neodecanoic acid (0.104 g, 0.6 mmol), Aliquat HTA-1 35%wt in H_2O (0.452 g, 0.6 mmol) are weighted in the pressure-tight 10 mL screw-cap glass tube, then 2 mL of K-EL 2 wt%-toluene (9:1 v/v, 2 mL) oil-in-water emulsion are added. The reaction mixture is stirred and allowed to homogenize for 5 min at RT, subsequently it is heated under stirring at 130°C through oil bath for 24 h. The mixture is cooled, then diluted with 8 mL of methanol and filtered. The crude was purified by filtration on a pad of silica gel (15 g) with CH_2Cl_2 (30 mL). The product was recrystallized from ethanol (30 mL) and isolated as a white solid in 82% yield (505 mg, 0.82 mmol). Mp: 72-73 °C. ^1H NMR (400 MHz, $CDCl_3$) δ 7.47 – 7.41 (m, 2H), 7.37 – 7.29 (m, 6H), 2.70 (t, *J* = 7.6 Hz, 4H), 1.73 – 1.61 (m, 4H), 1.43 – 1.27 (m, 12H), 0.90 (t, *J* = 7.1 Hz, 6H). ^{13}C NMR (100 MHz, $CDCl_3$): δ 144.4 (dd, J=251.6, 16.7 Hz), 144.1 (d, J=243.5 Hz), 143.5, 130.1, 129.7, 128.6, 127.3, 126.8, 122.9 (t, J=16.7 Hz), 105.9 (m), 35.9, 31.7, 31.3, 28.9, 22.6, 14.1. Anal. Calcd for $C_{36}H_{34}F_8$: C, 69.89; H, 5.54. Found: C, 70.03; H, 5.64.

E-factor calculation

$$E_{factor} = \frac{mass\ of\ reagents + mass\ of\ auxiliaries - mass\ of\ product}{mass\ of\ product} =$$
$$= \frac{3.6364 + 84.906 - 0.505}{0.505} = 174$$

2 UV-Vis absorption and PL spectra for derivatives 1, 8-15

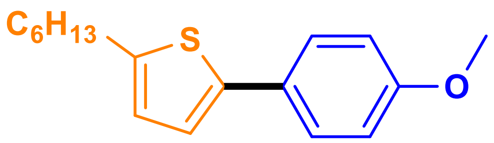

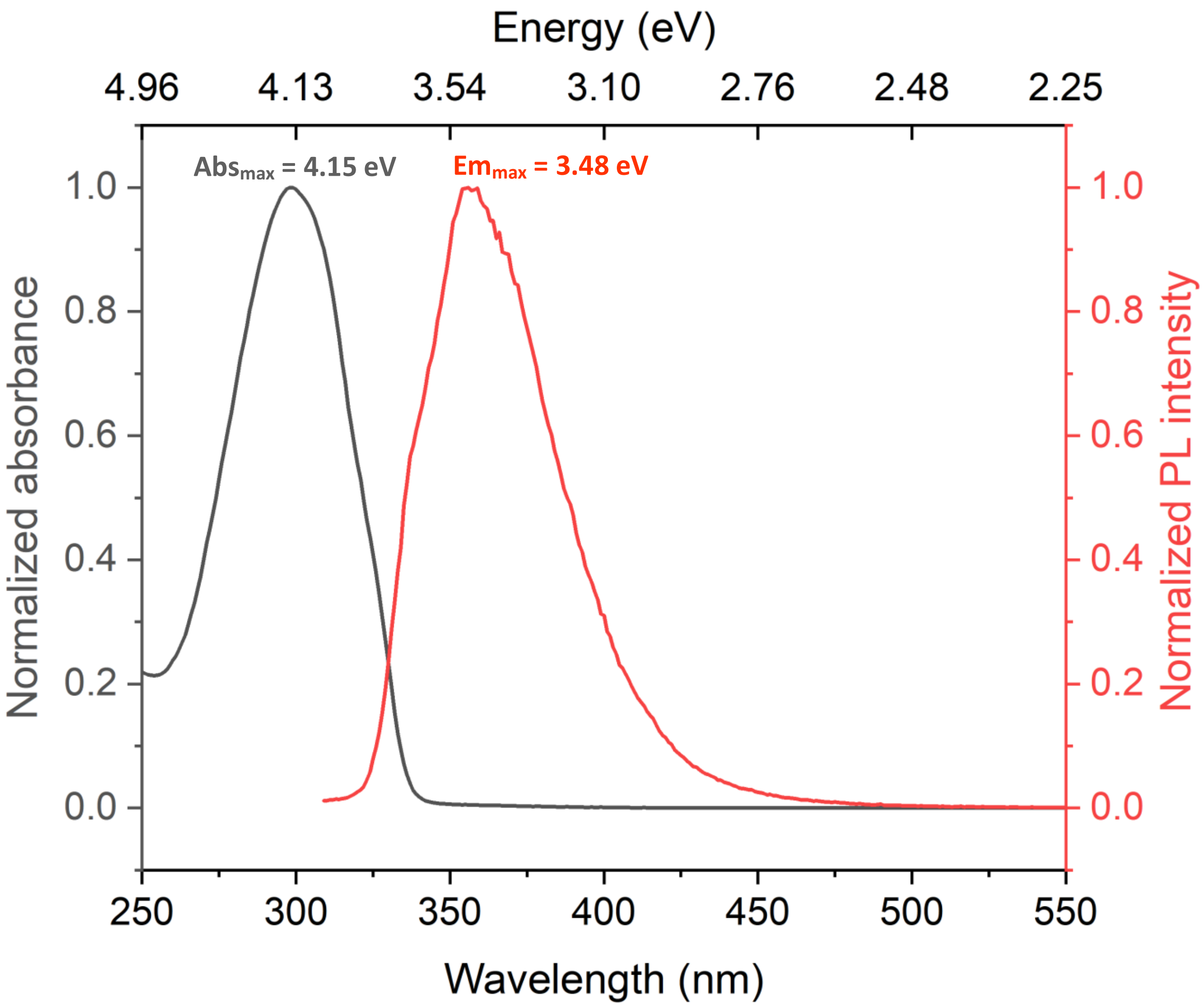

Stokes shift = 0.67 eV.

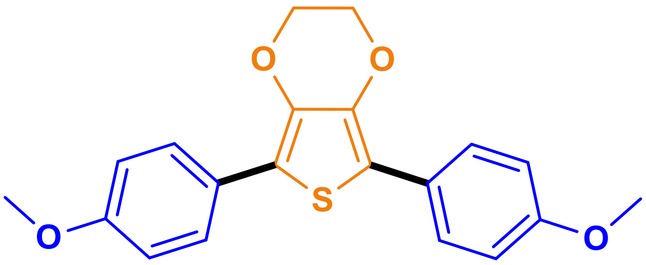

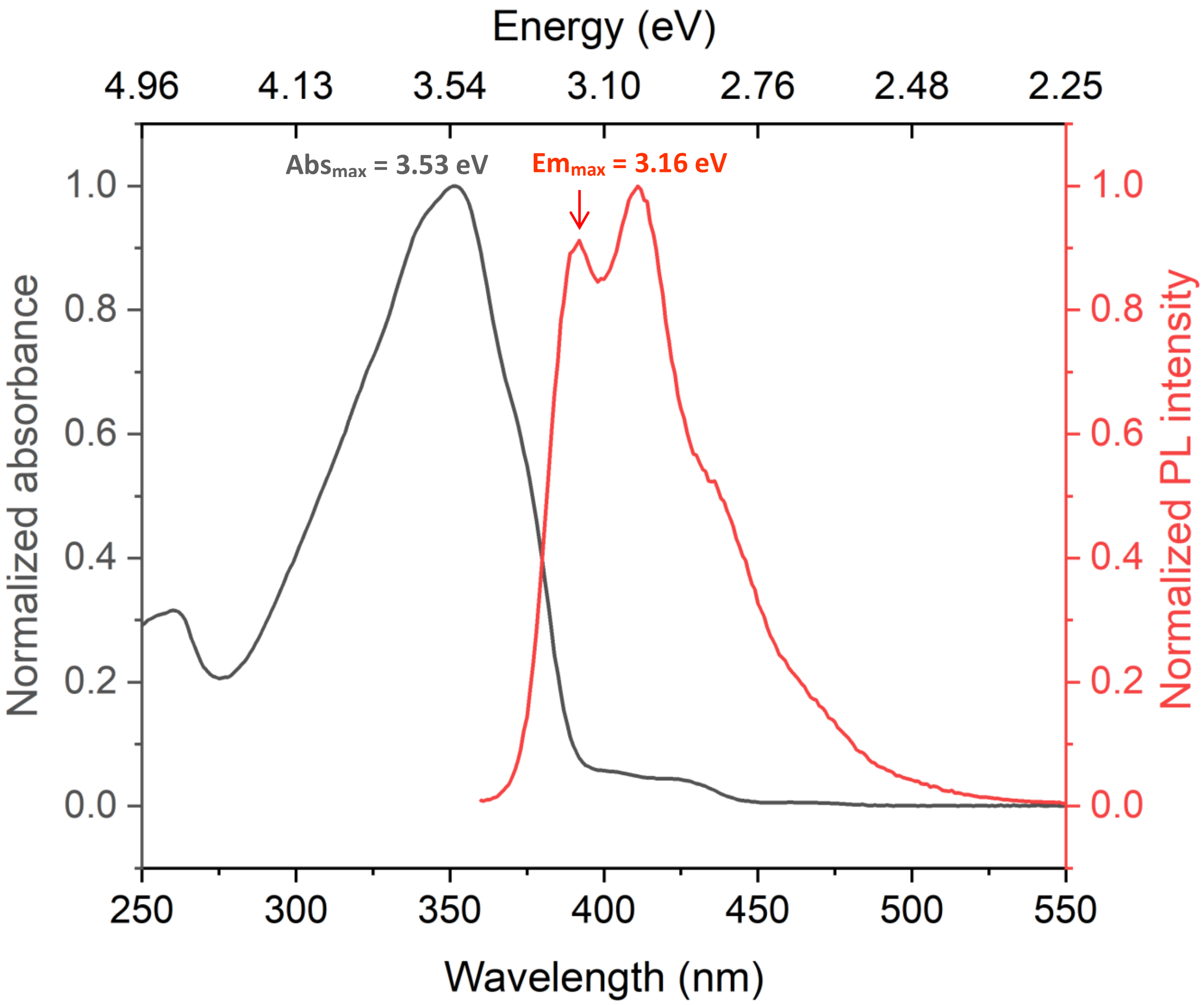

Stokes shift = 0.37 eV.

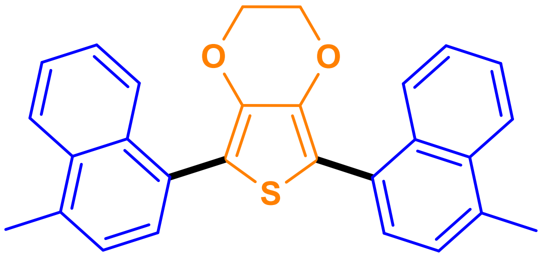

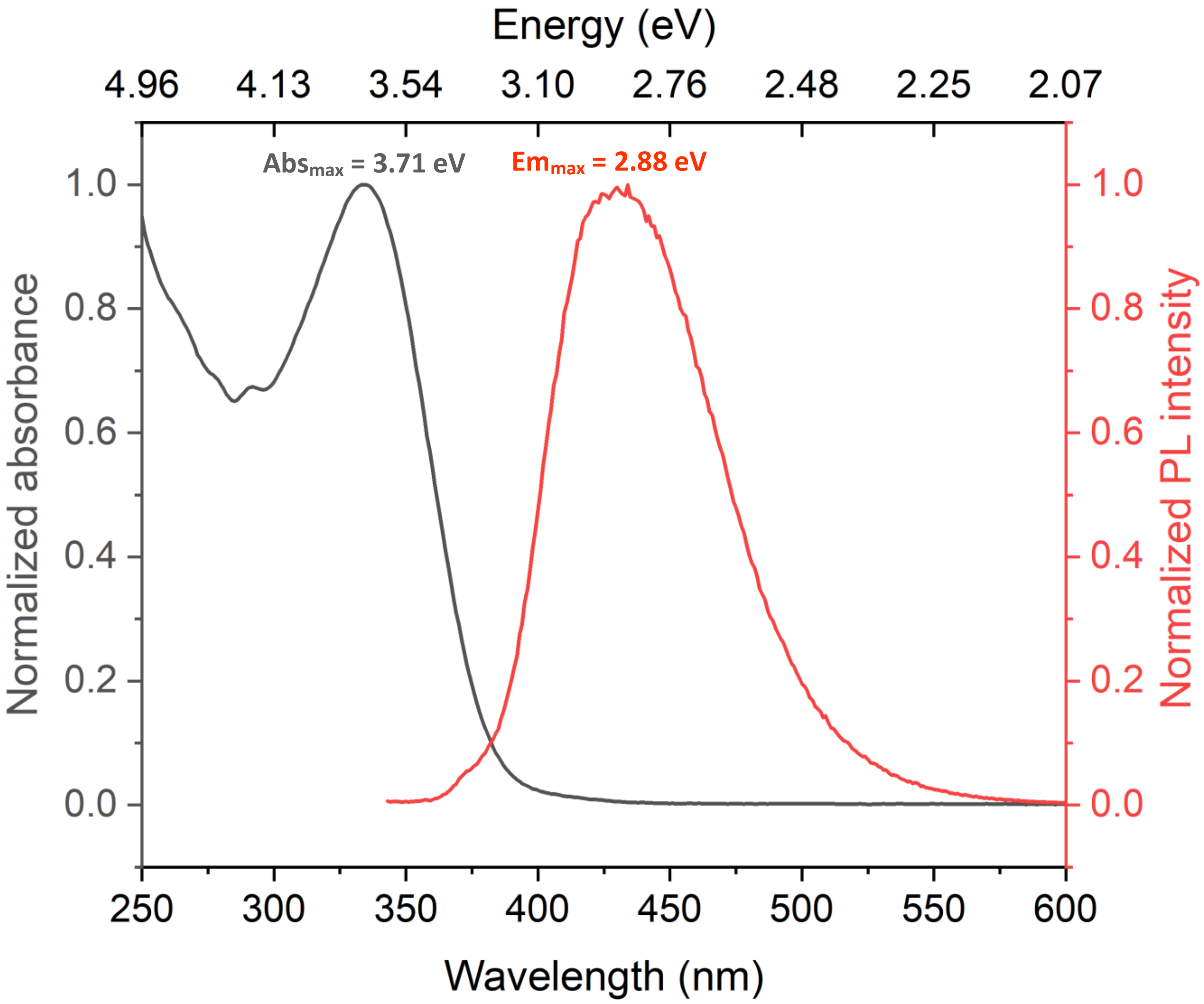

Stokes shift = 0.83 eV.

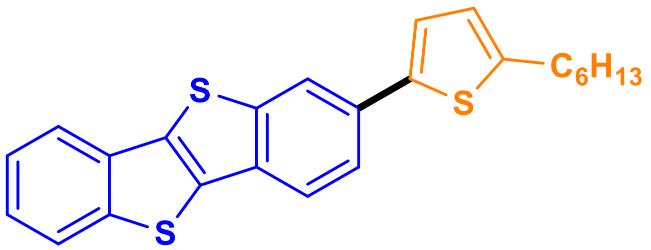

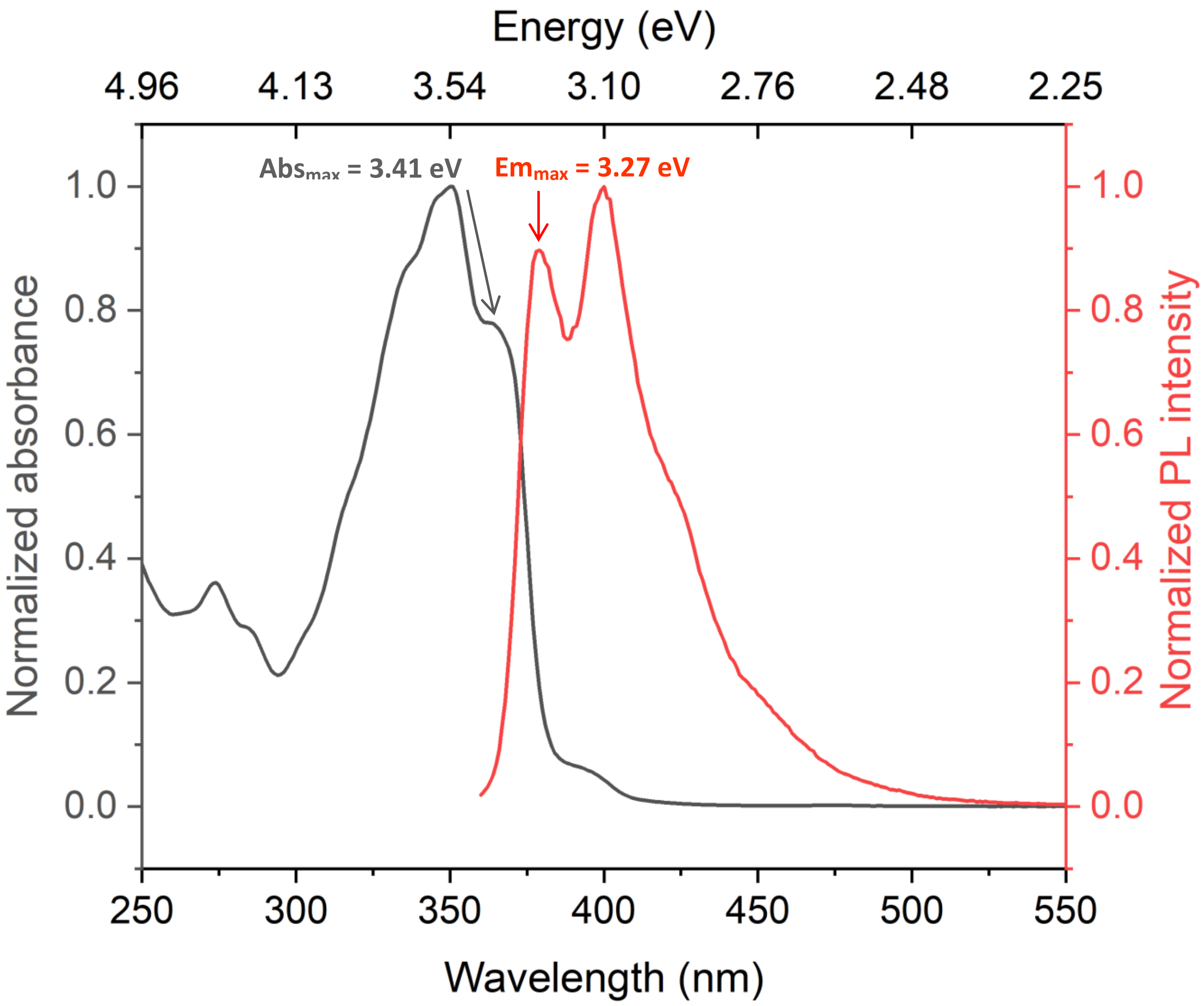

Stokes shift = 0.14 eV.

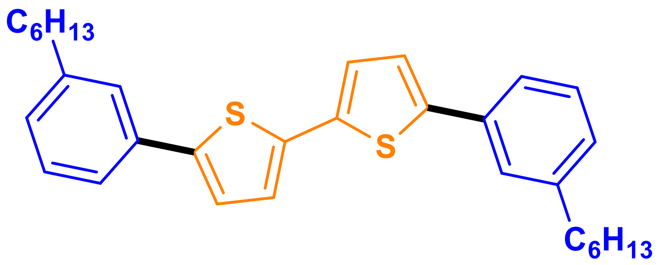

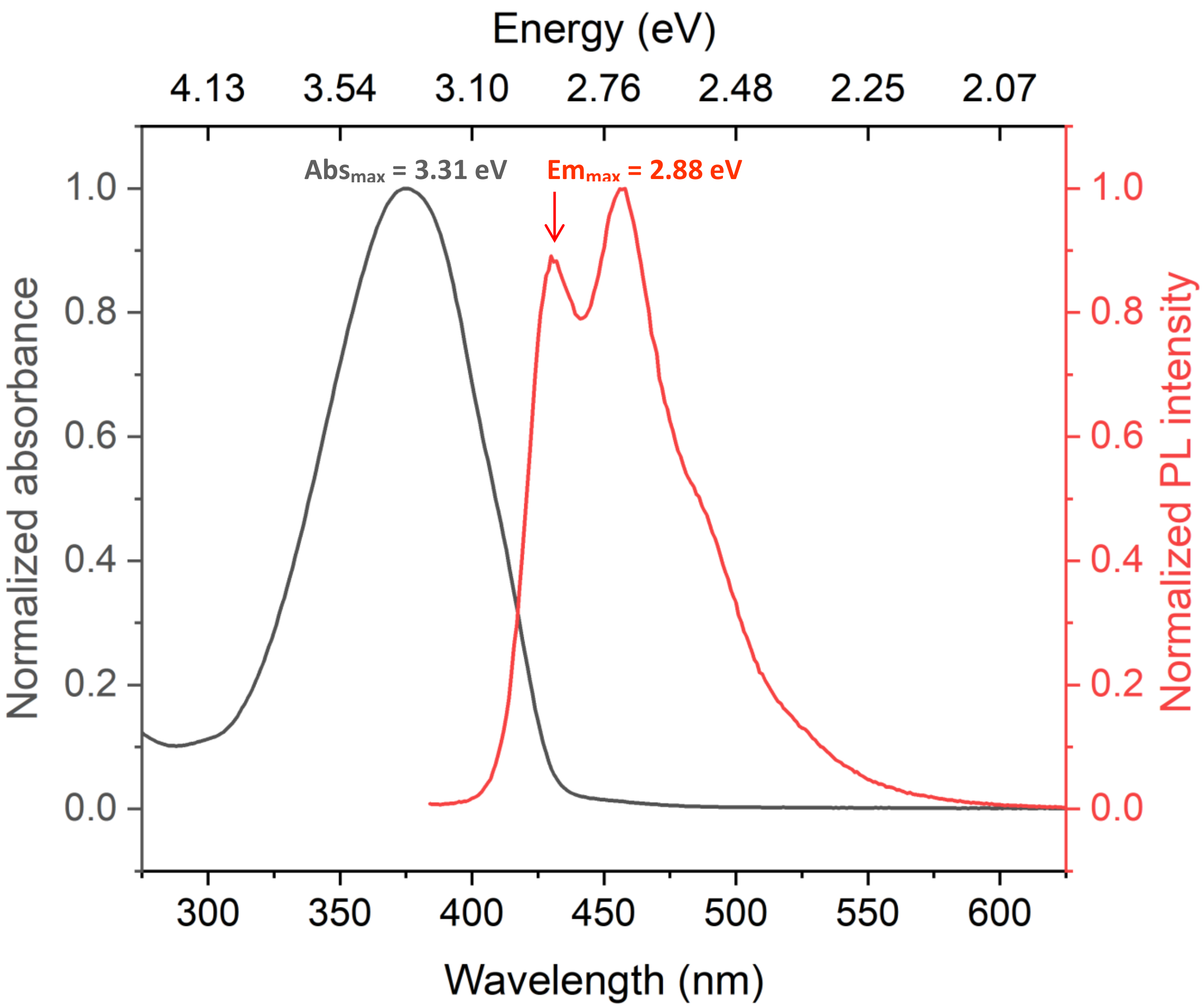

Stokes shift = 0.43 eV.

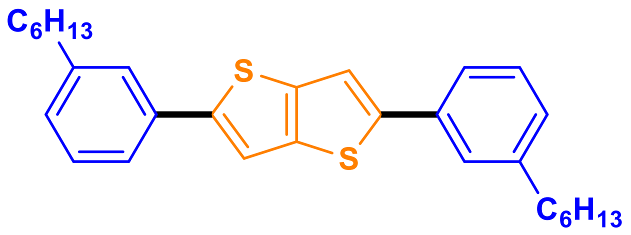

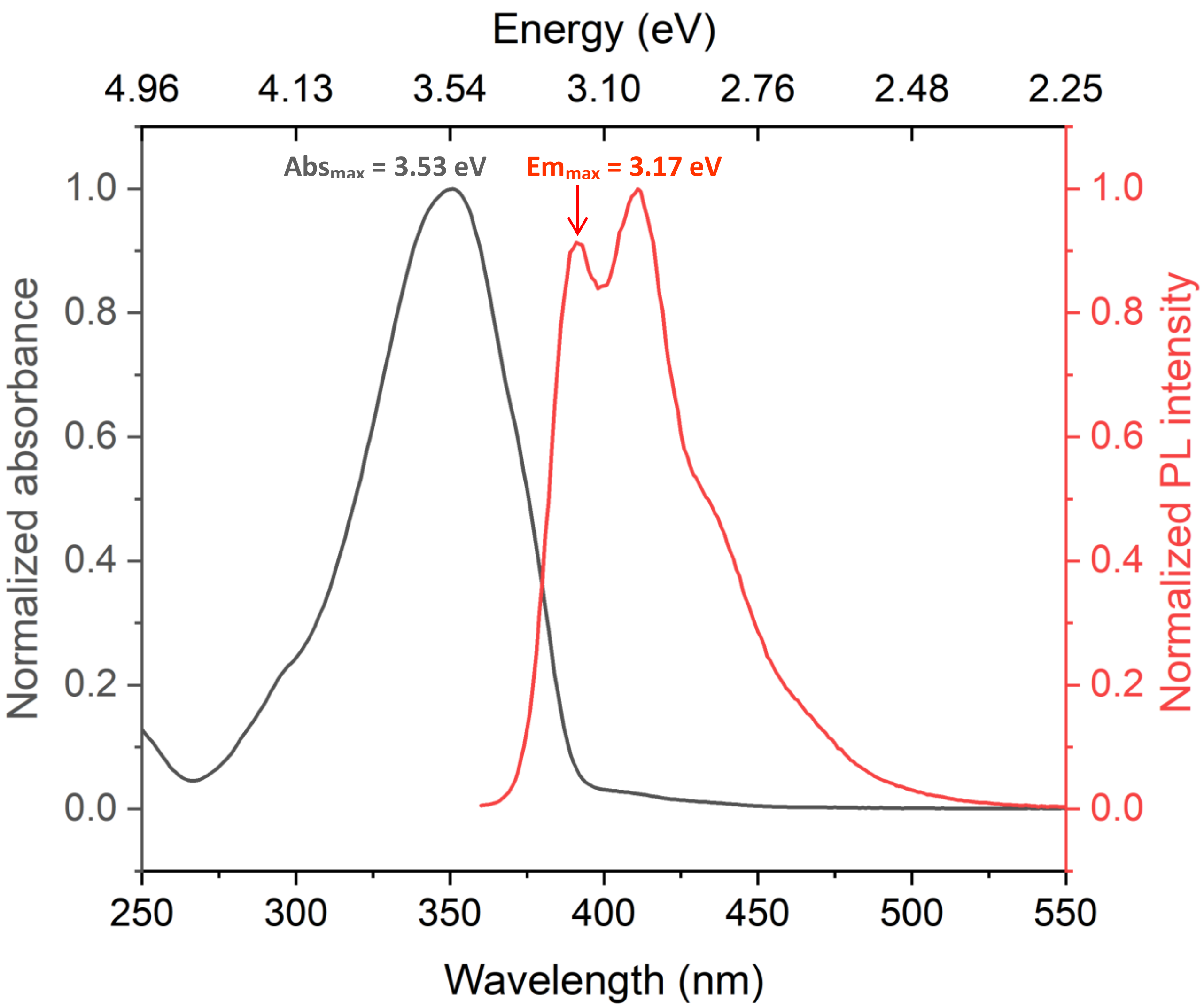

Stokes shift = 0.36 eV.

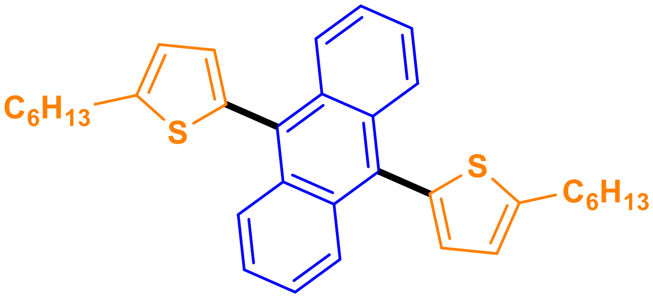

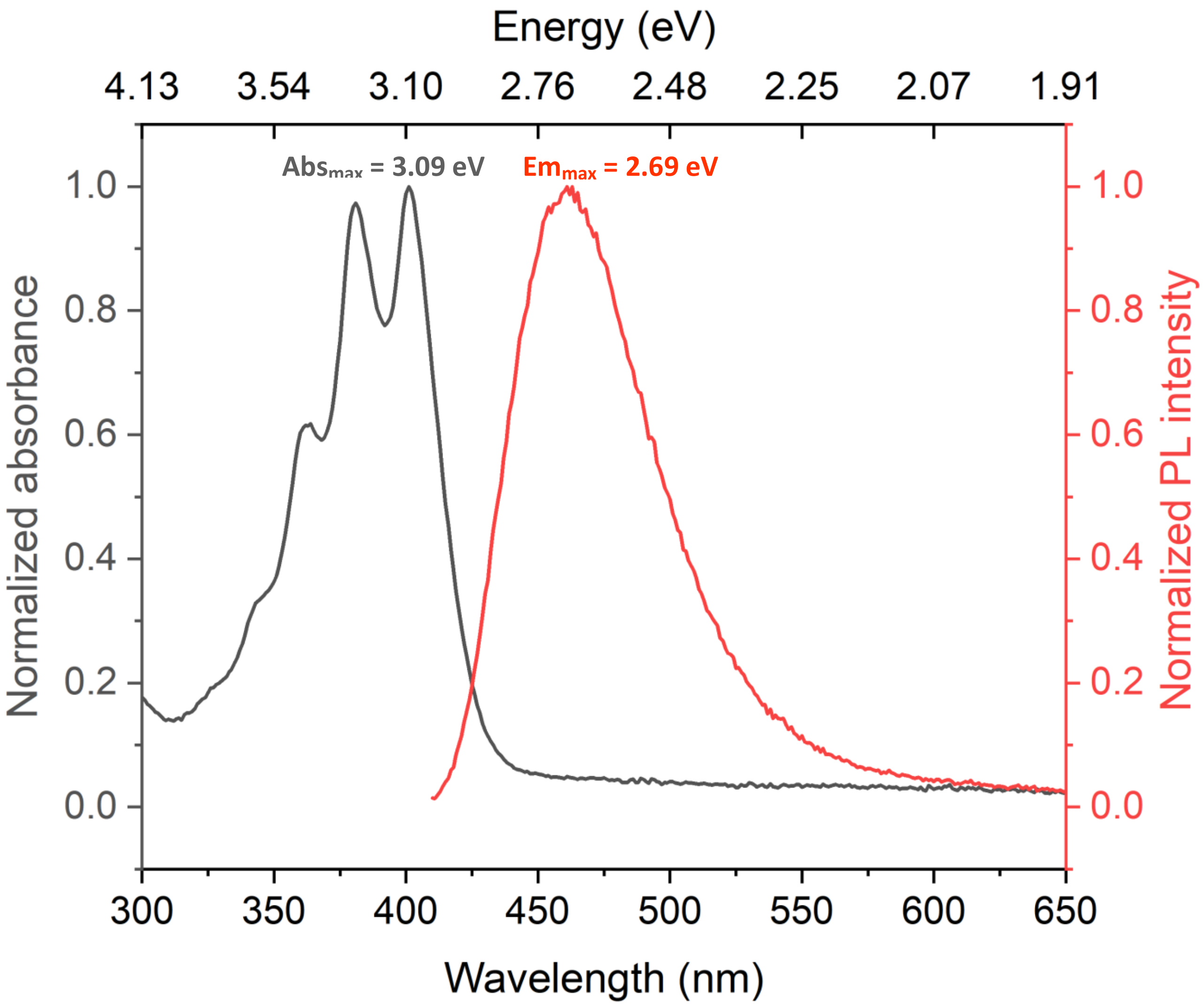

Stokes shift = 0.40 eV.

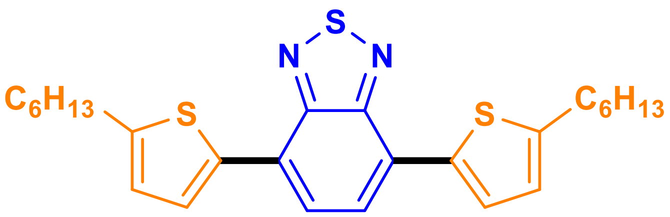

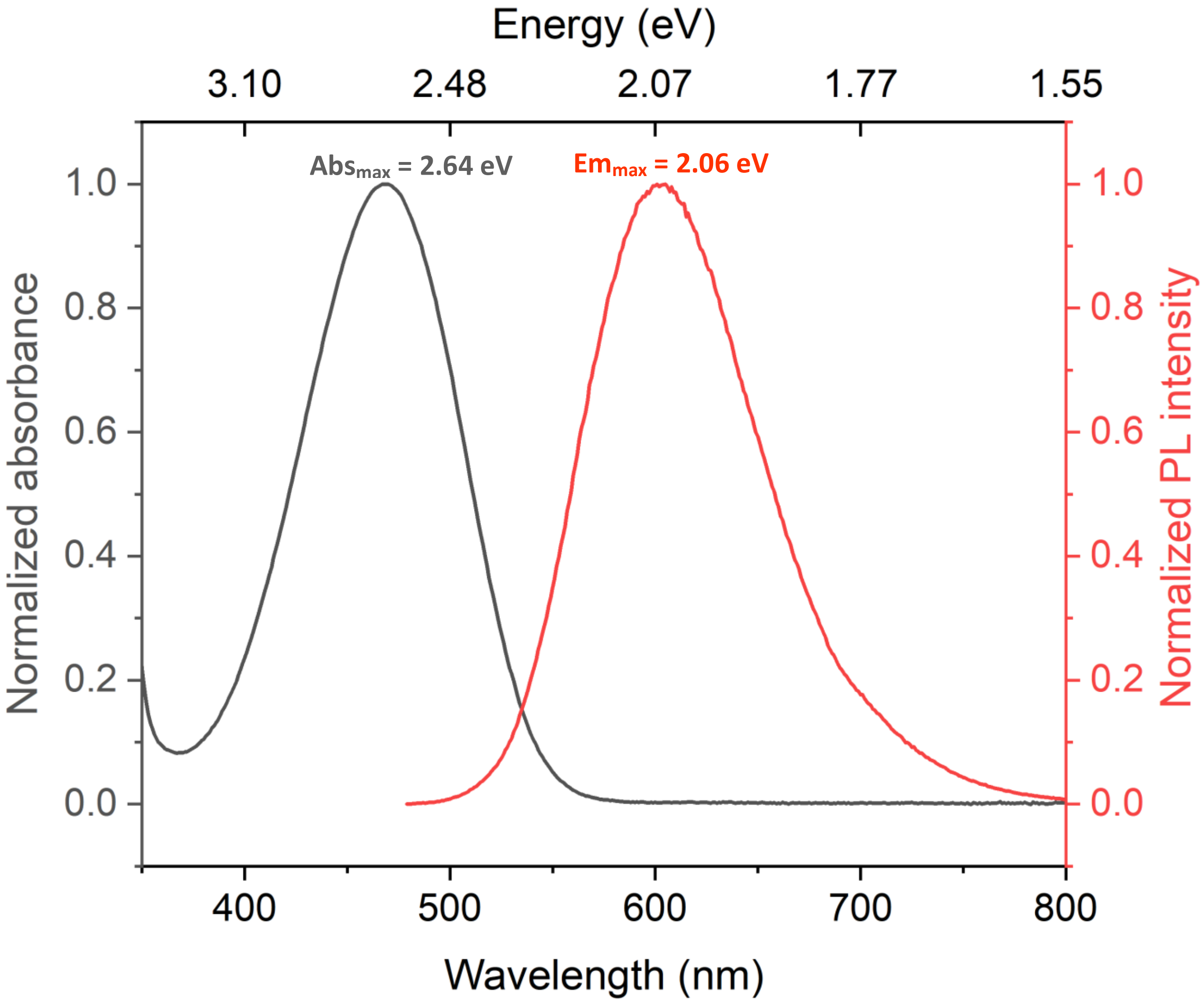

Stokes shift = 0.58 eV.

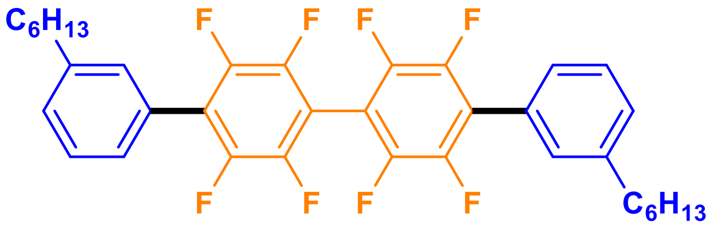

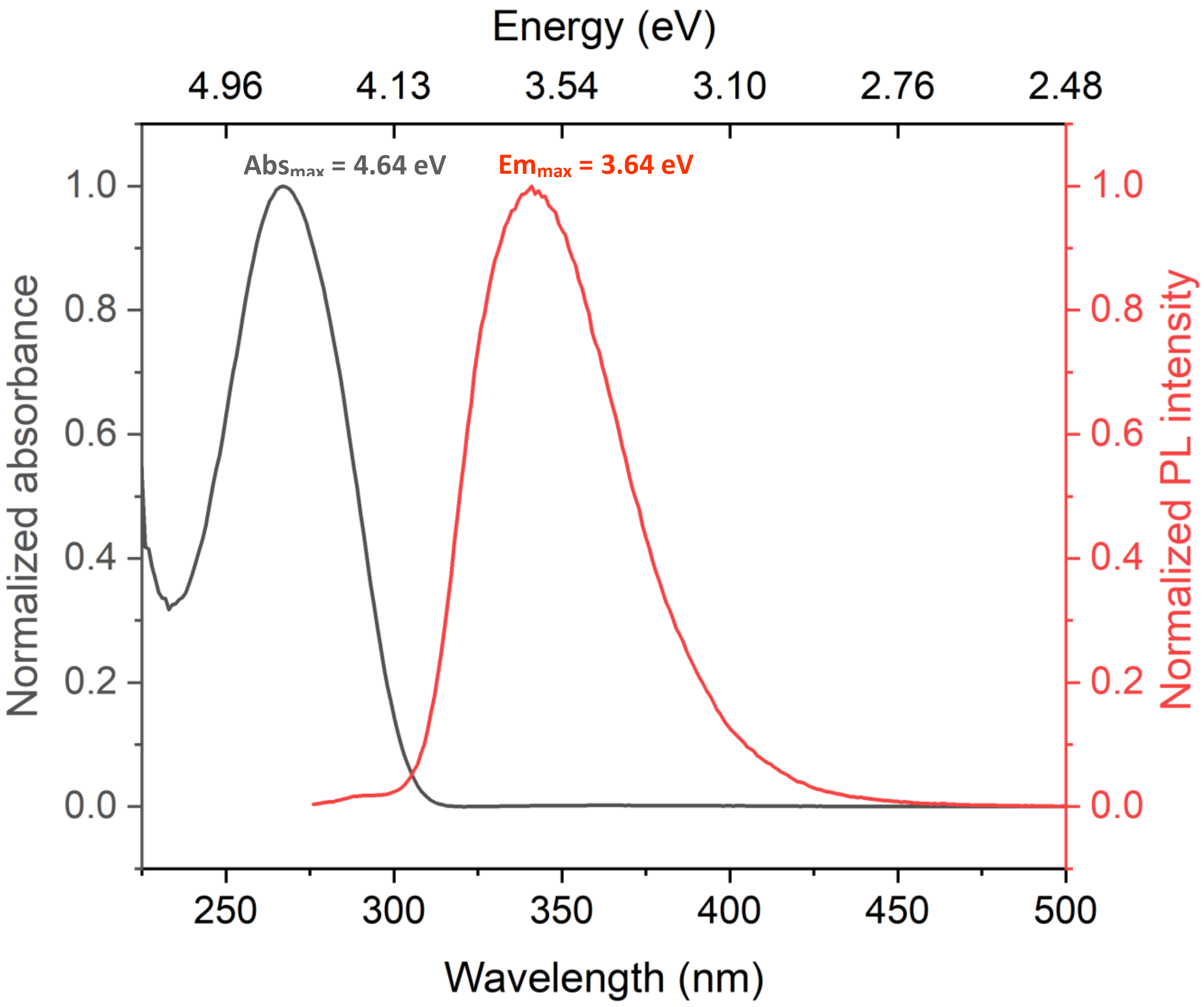

Stokes shift = 1.00 eV.

3 DPV plots of derivatives 8-15

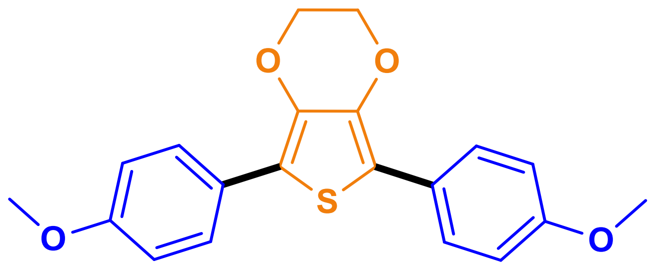

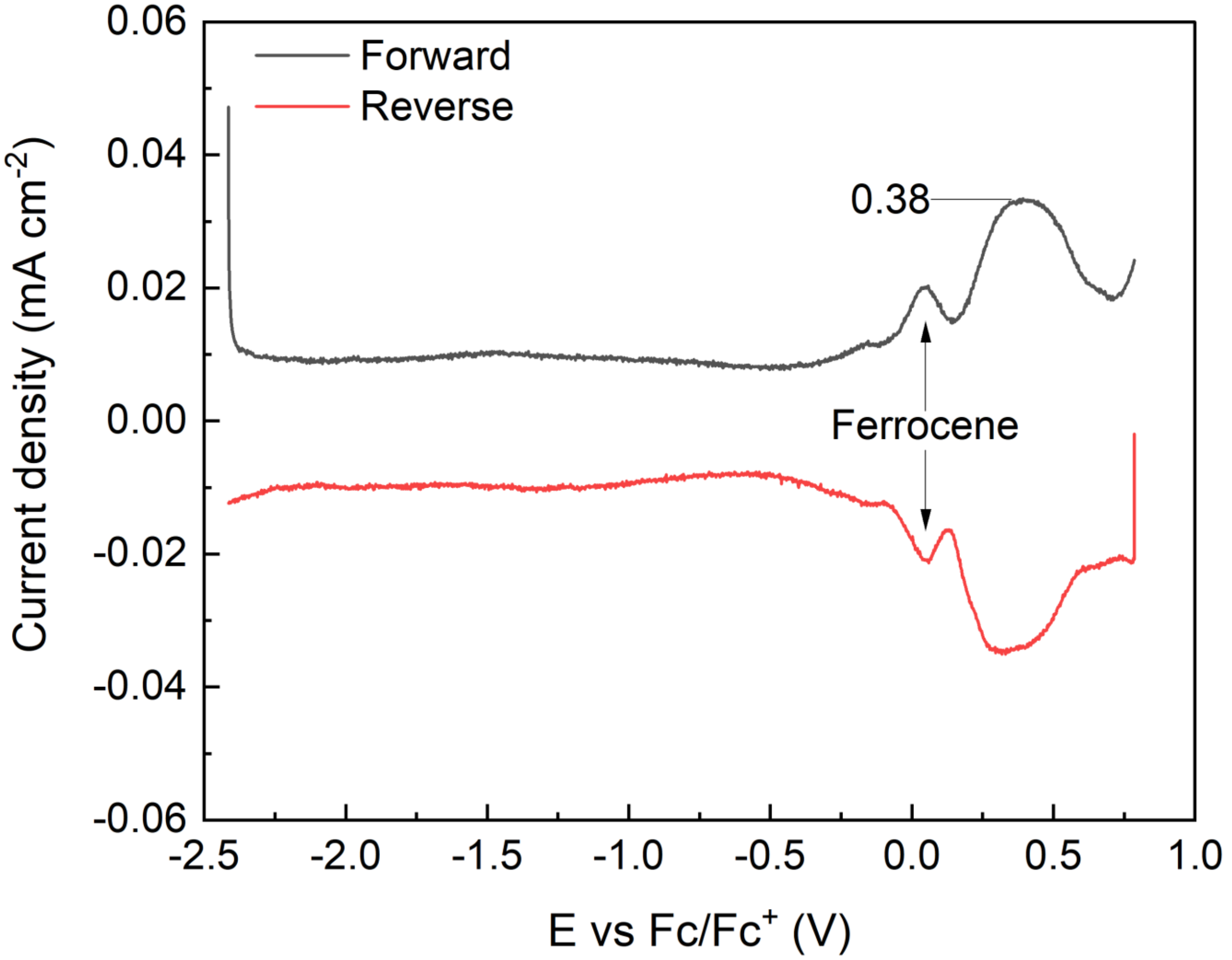

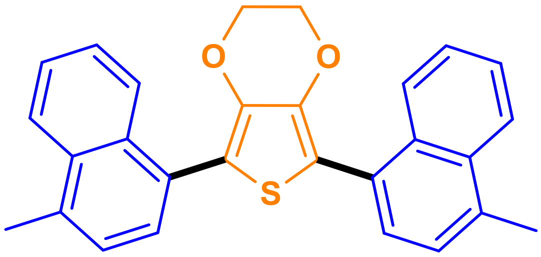
O
O
S

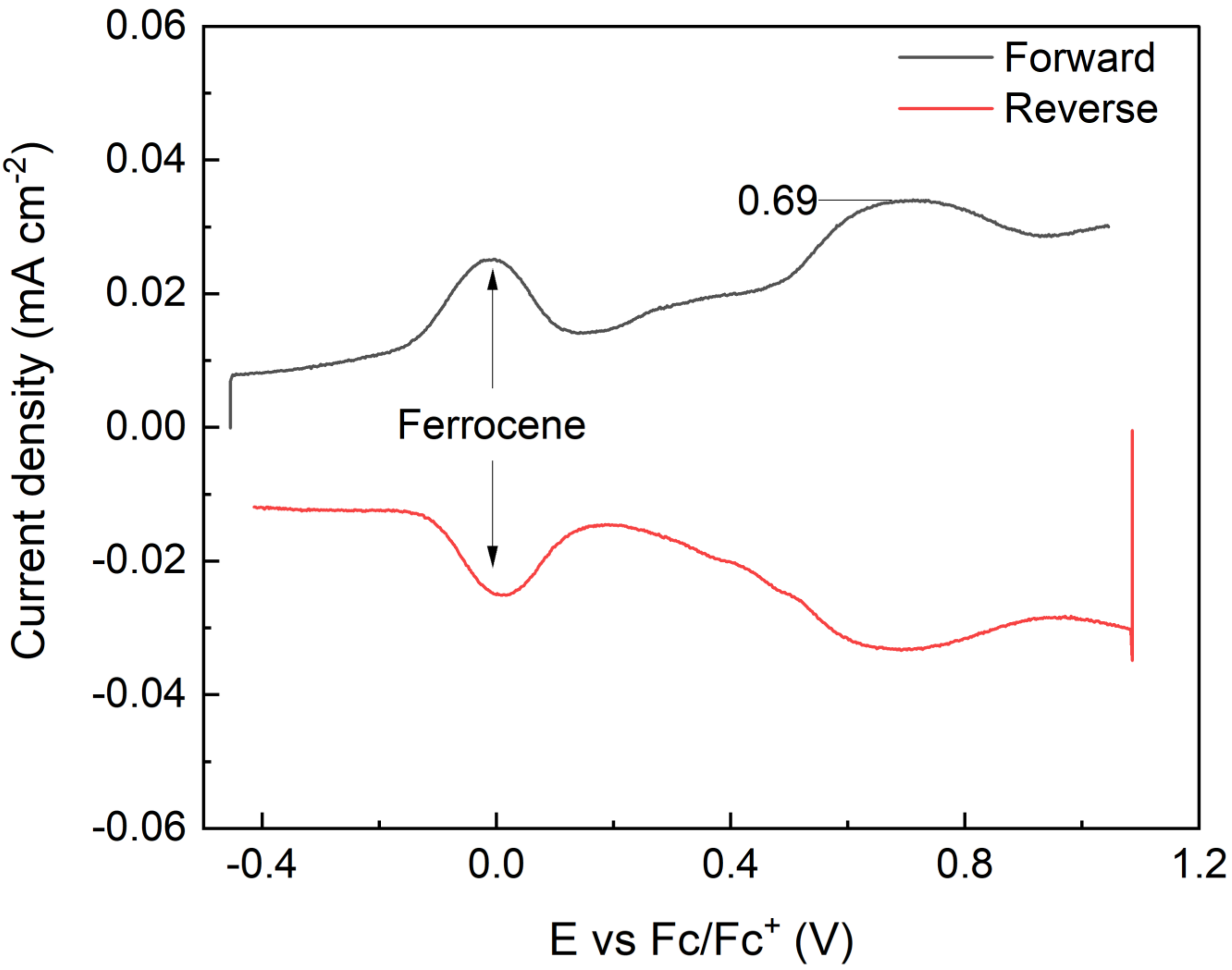
Forward
Reverse
0.69
Ferrocene
Current density (mA cm^{-2})
E vs Fc/Fc^{+} (V)
0.06
0.04
0.02
0.00
-0.02
-0.04
-0.06
-0.4
0.0
0.4
0.8
1.2

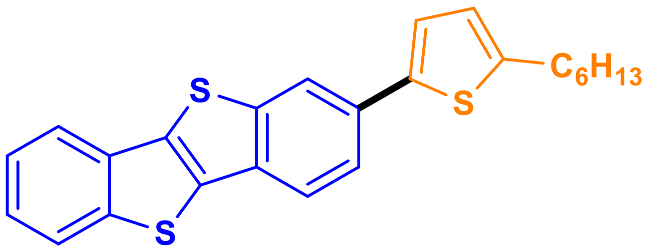
S
S
S
C6H13

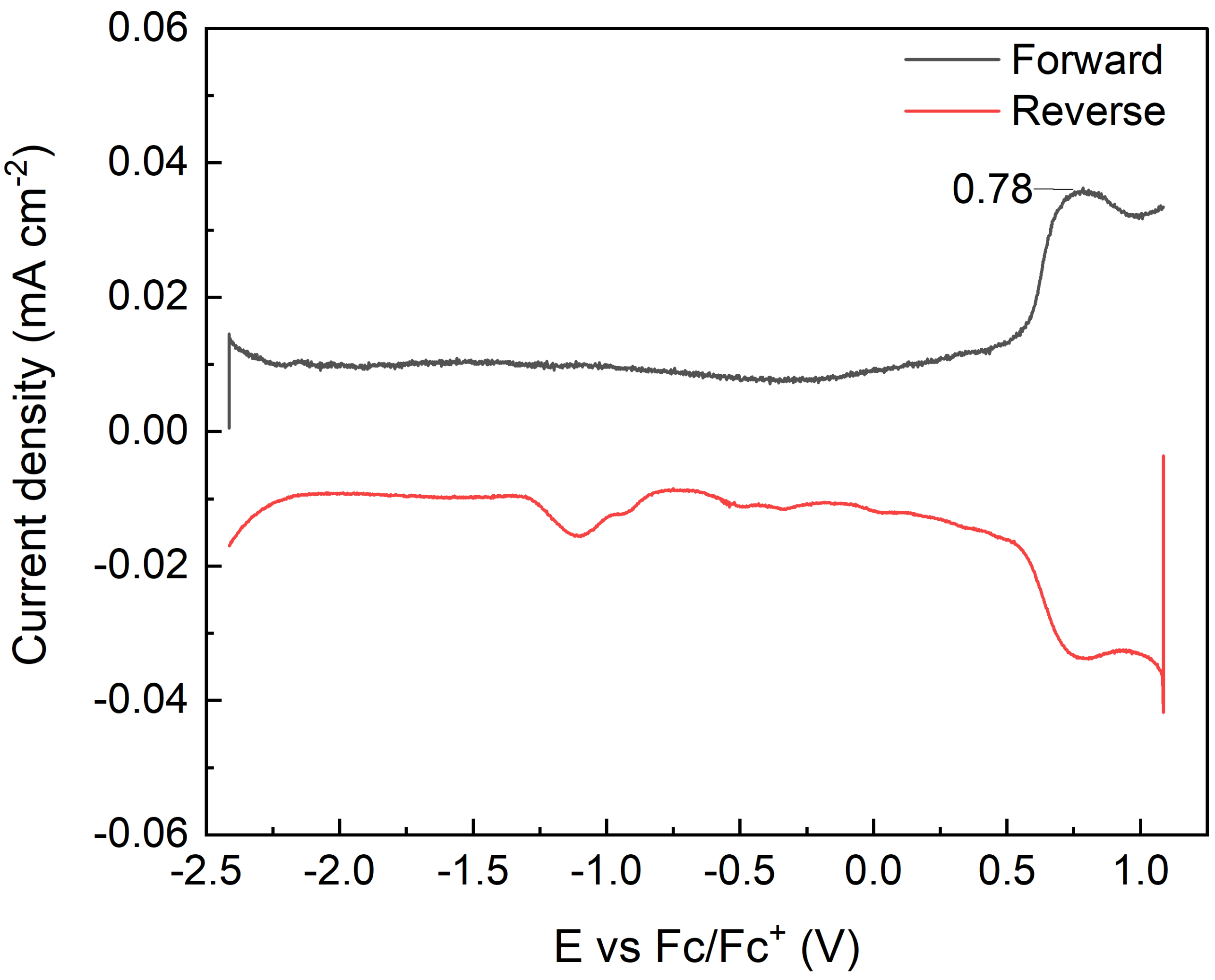
0.06
0.04
0.02
0.00
-0.02
-0.04
-0.06
Current density (mA cm-2)
-2.5
-2.0
-1.5
-1.0
-0.5
0.0
0.5
1.0
E vs Fc/Fc+ (V)
Forward
Reverse
0.78

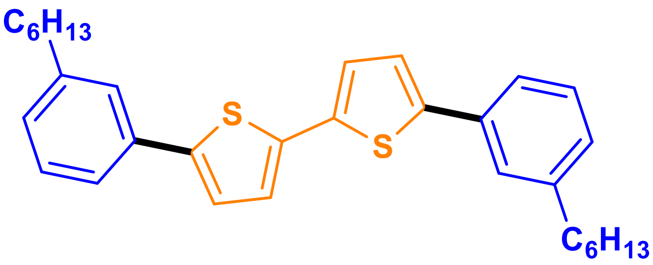

C6H13
S
S
C6H13

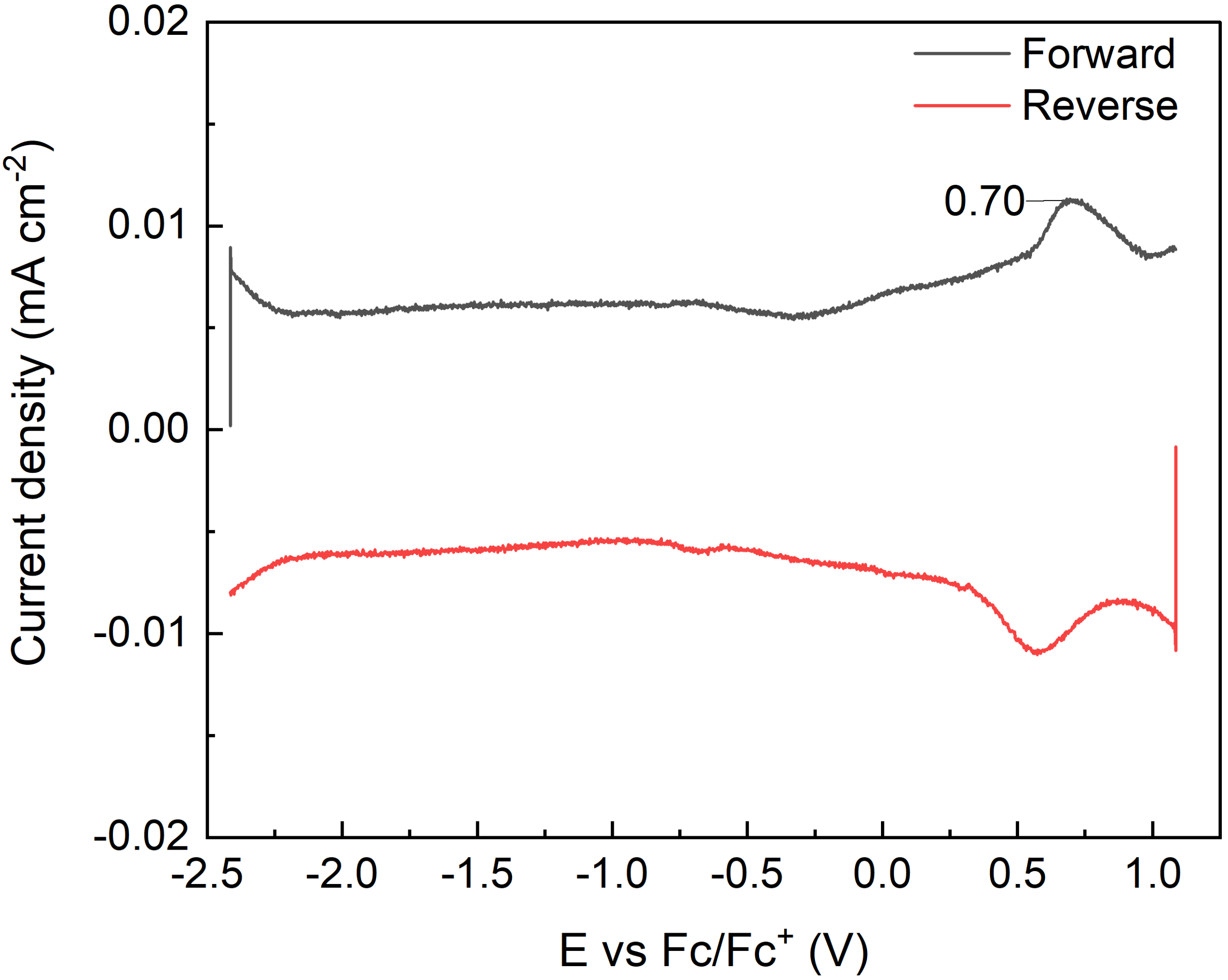

Forward
Reverse
0.70
Current density (mA cm-2)
E vs Fc/Fc+ (V)
0.02
0.01
0.00
-0.01
-0.02
-2.5
-2.0
-1.5
-1.0
-0.5
0.0
0.5
1.0

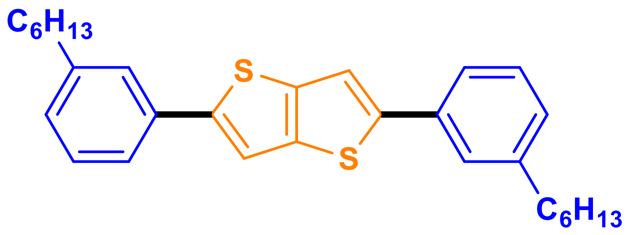
C6H13
S
S
C6H13

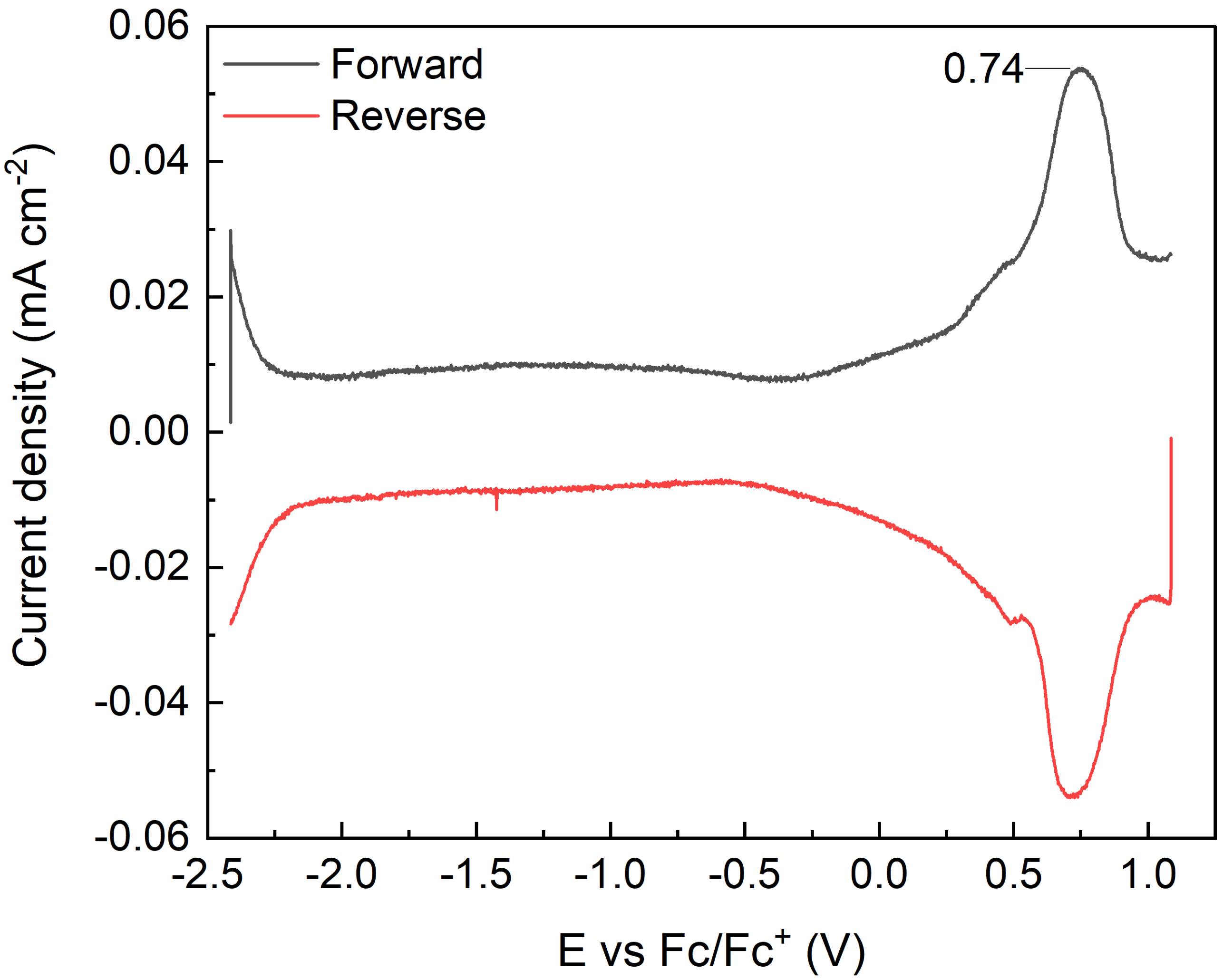
Forward
Reverse
0.74
Current density (mA cm-2)
E vs Fc/Fc+ (V)
0.06
0.04
0.02
0.00
-0.02
-0.04
-0.06
-2.5
-2.0
-1.5
-1.0
-0.5
0.0
0.5
1.0

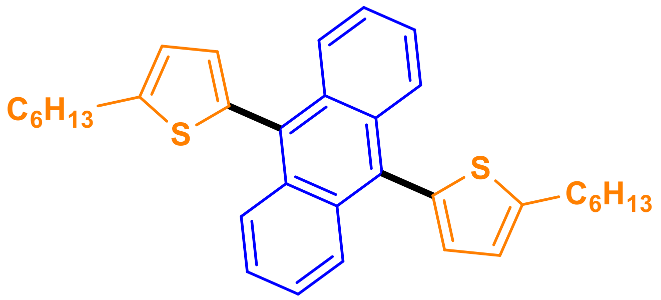
C6H13
S
S
C6H13

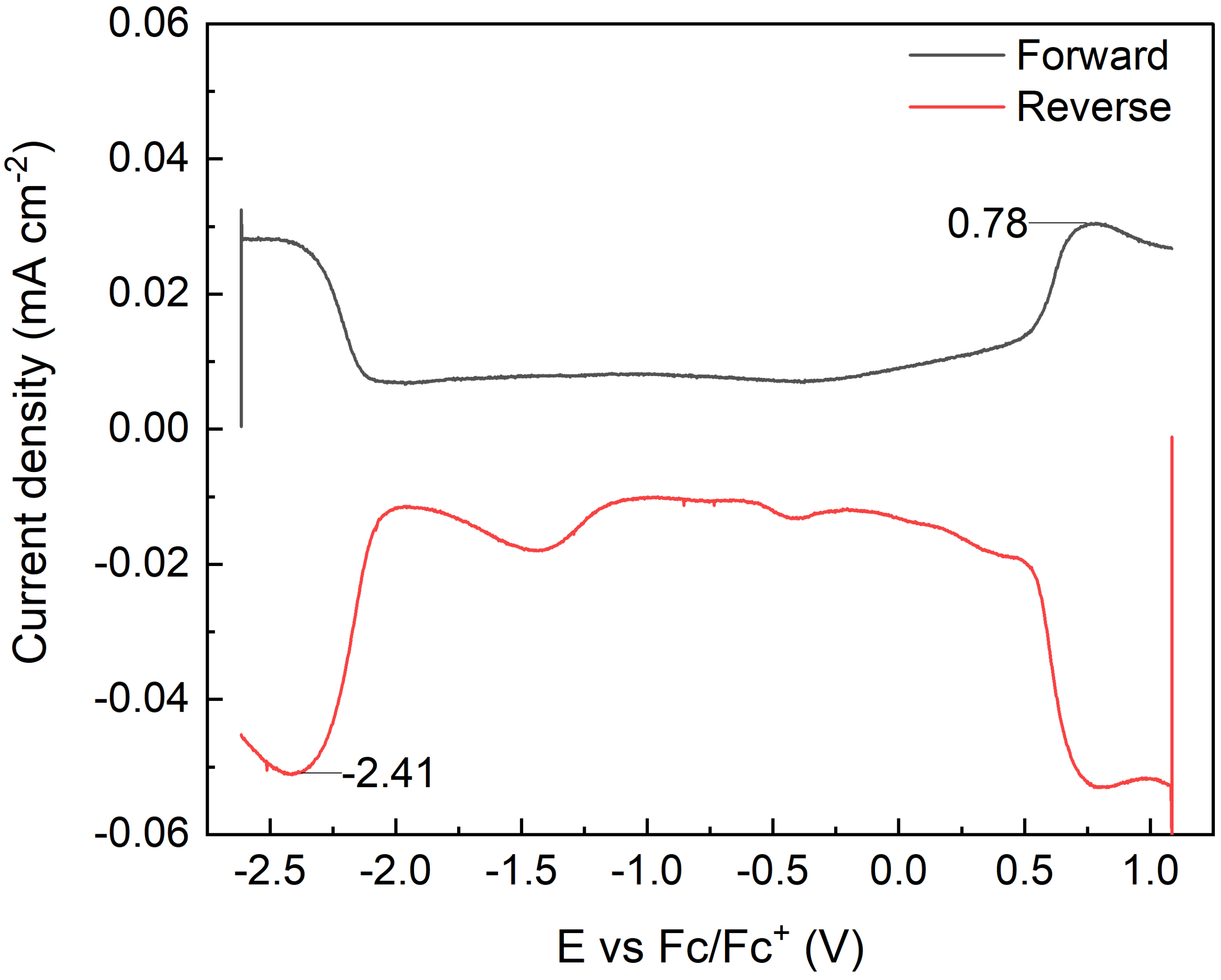
Forward
Reverse
0.78
-2.41
Current density (mA cm-2)
E vs Fc/Fc+ (V)
0.06
0.04
0.02
0.00
-0.02
-0.04
-0.06
-2.5
-2.0
-1.5
-1.0
-0.5
0.0
0.5
1.0

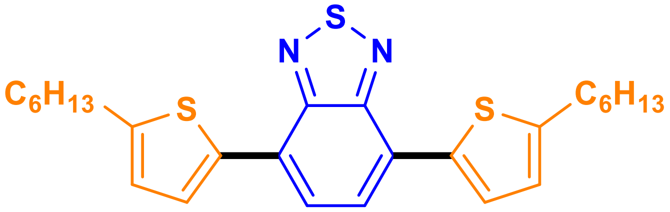
C6H13
S
N
S
N
S
C6H13

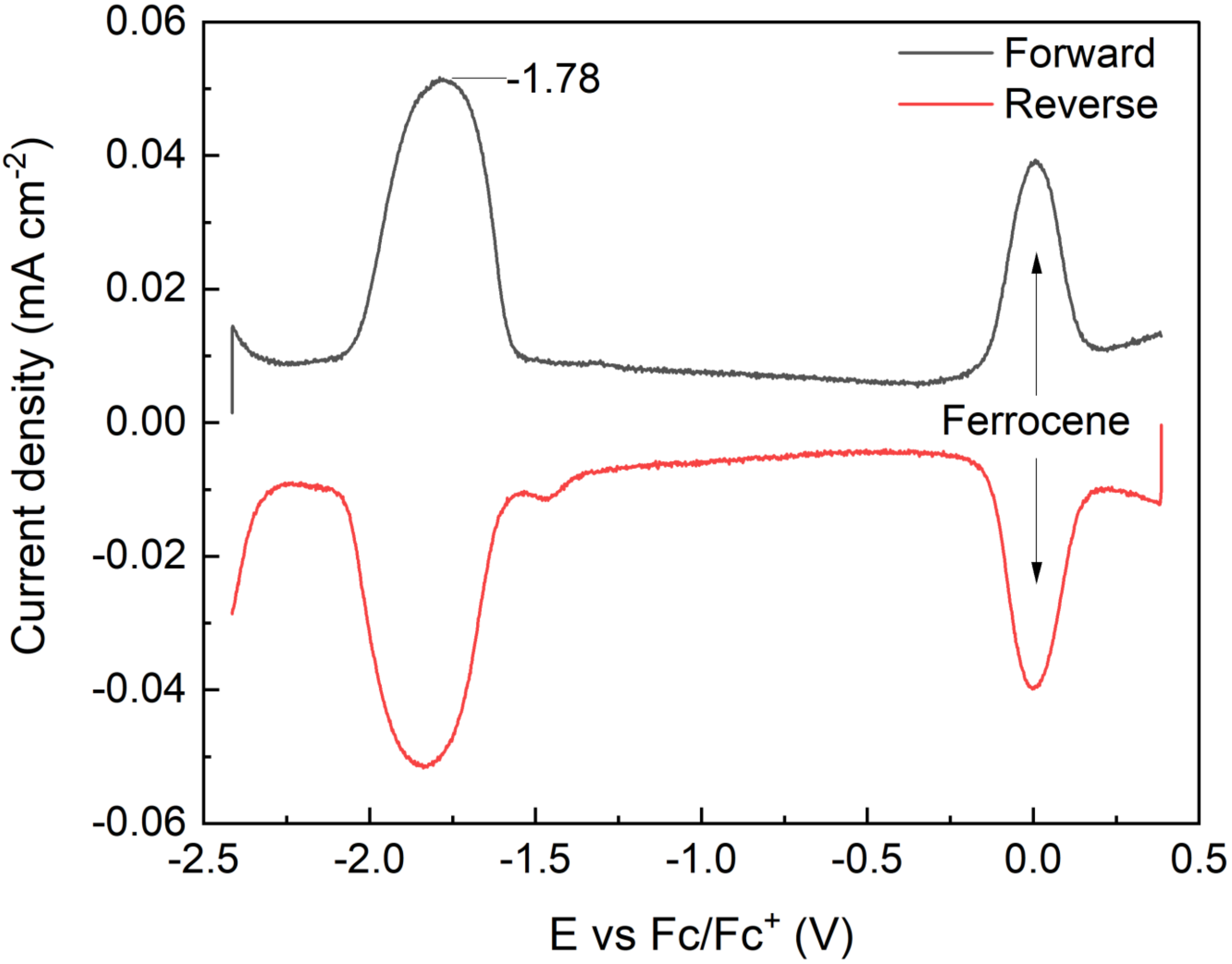
Forward
Reverse
-1.78
Ferrocene
Current density (mA cm-2)
E vs Fc/Fc+ (V)
0.06
0.04
0.02
0.00
-0.02
-0.04
-0.06
-2.5
-2.0
-1.5
-1.0
-0.5
0.0
0.5

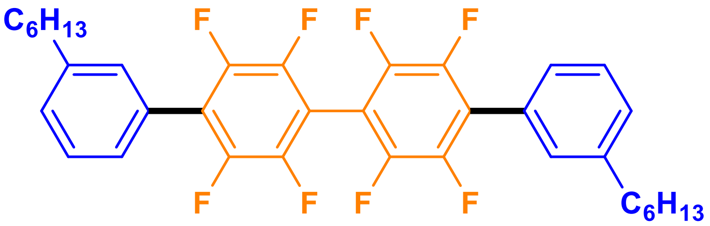

C6H13
F
F
F
F
F
F
F
F
C6H13

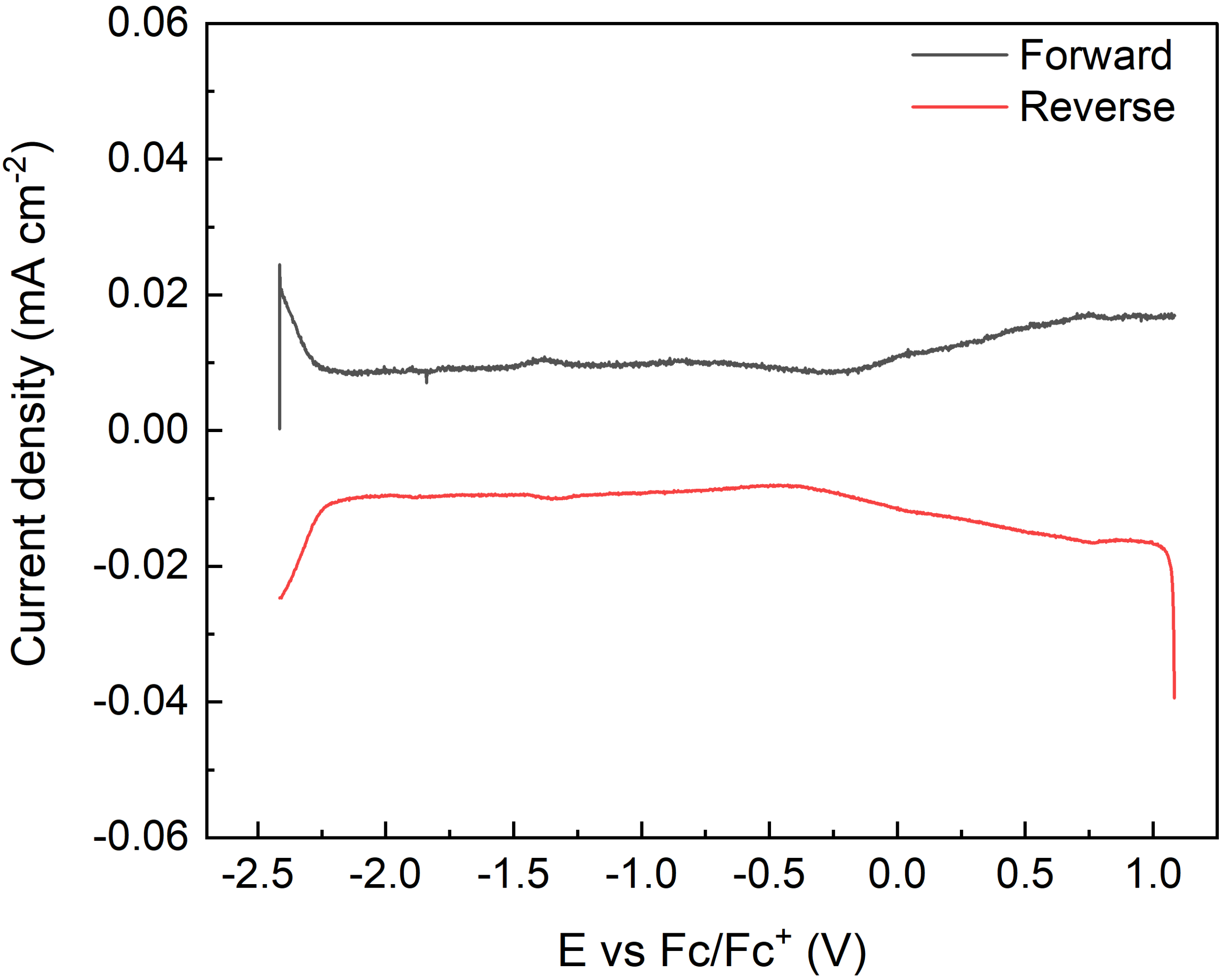

Forward
Reverse
Current density (mA cm-2)
E vs Fc/Fc+ (V)
0.06
0.04
0.02
0.00
-0.02
-0.04
-0.06
-2.5
-2.0
-1.5
-1.0
-0.5
0.0
0.5
1.0

4 ^{1}H and ^{13}C NMR spectra for all new and known derivatives

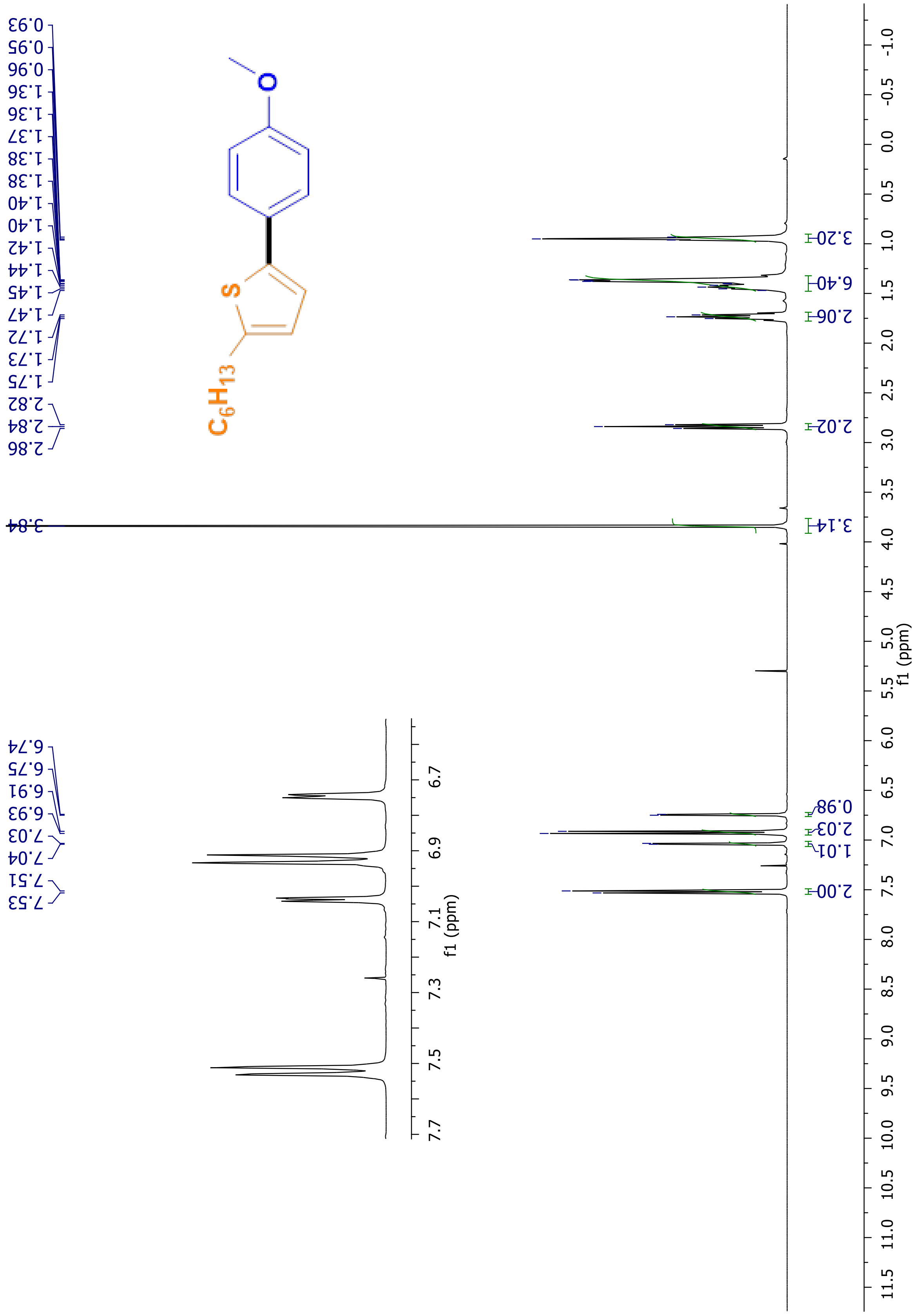

^{1}H NMR spectrum of derivative 1.

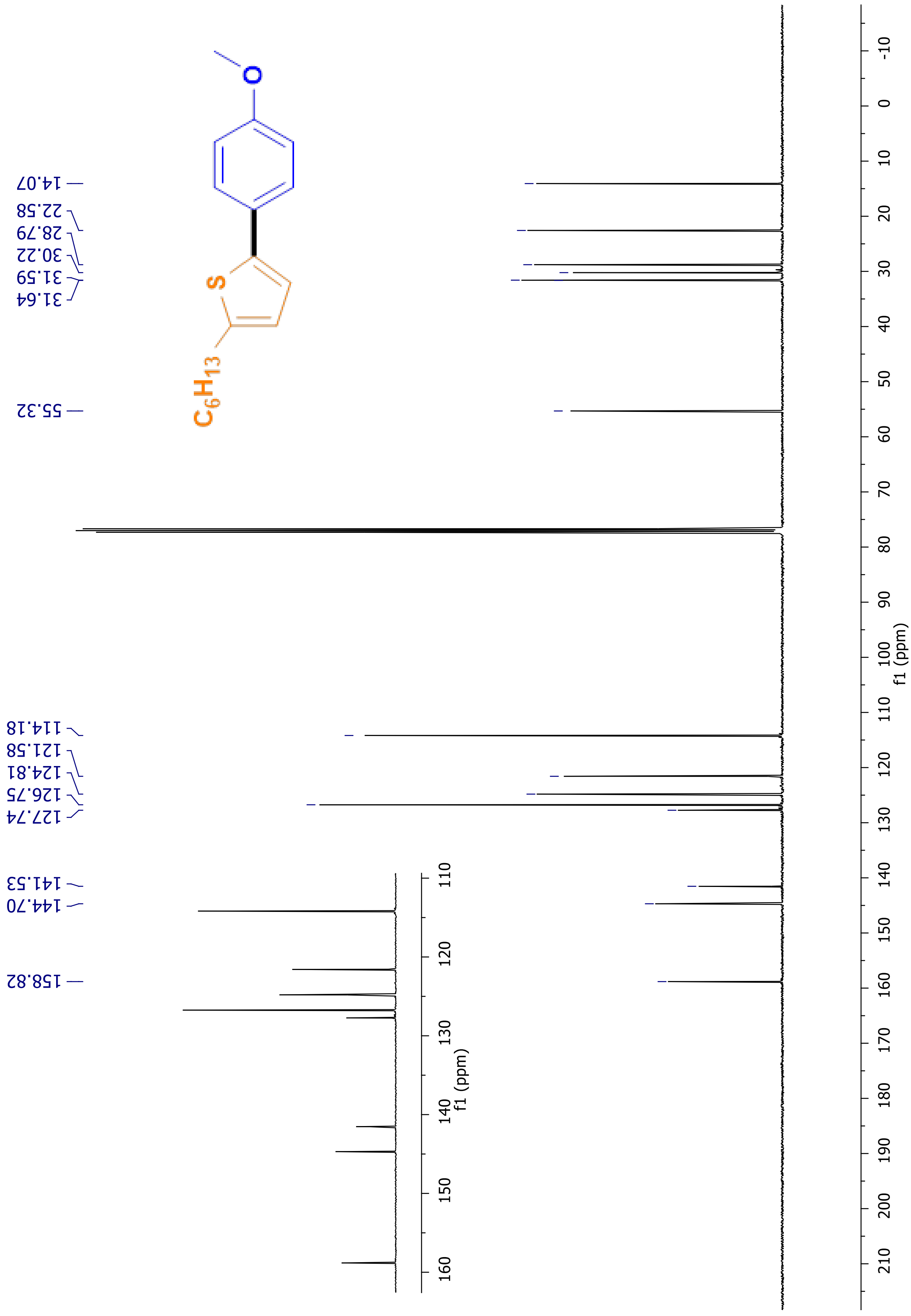

^{13}C NMR spectrum of derivative 1.

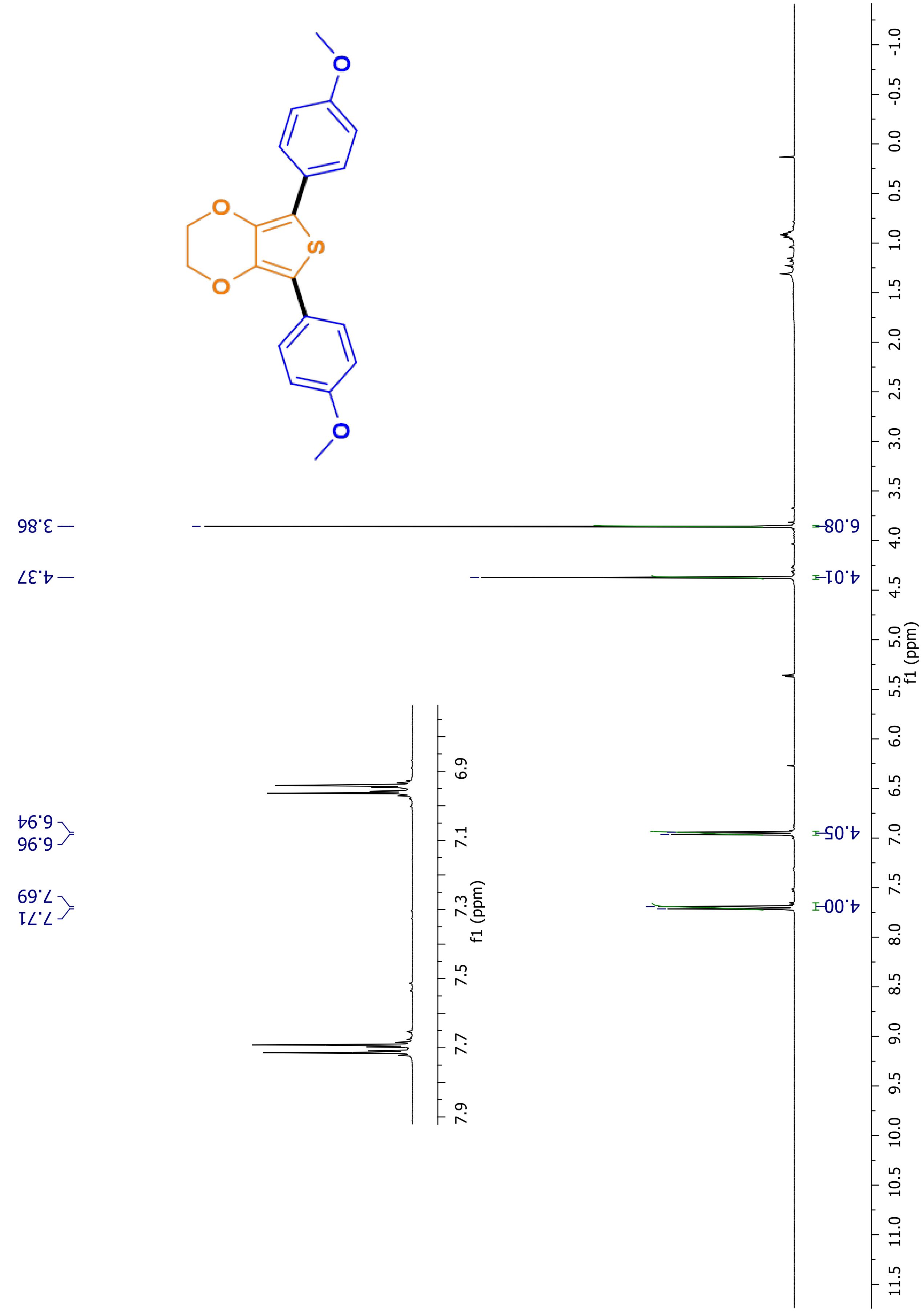

^{1}H NMR spectrum of derivative 8.

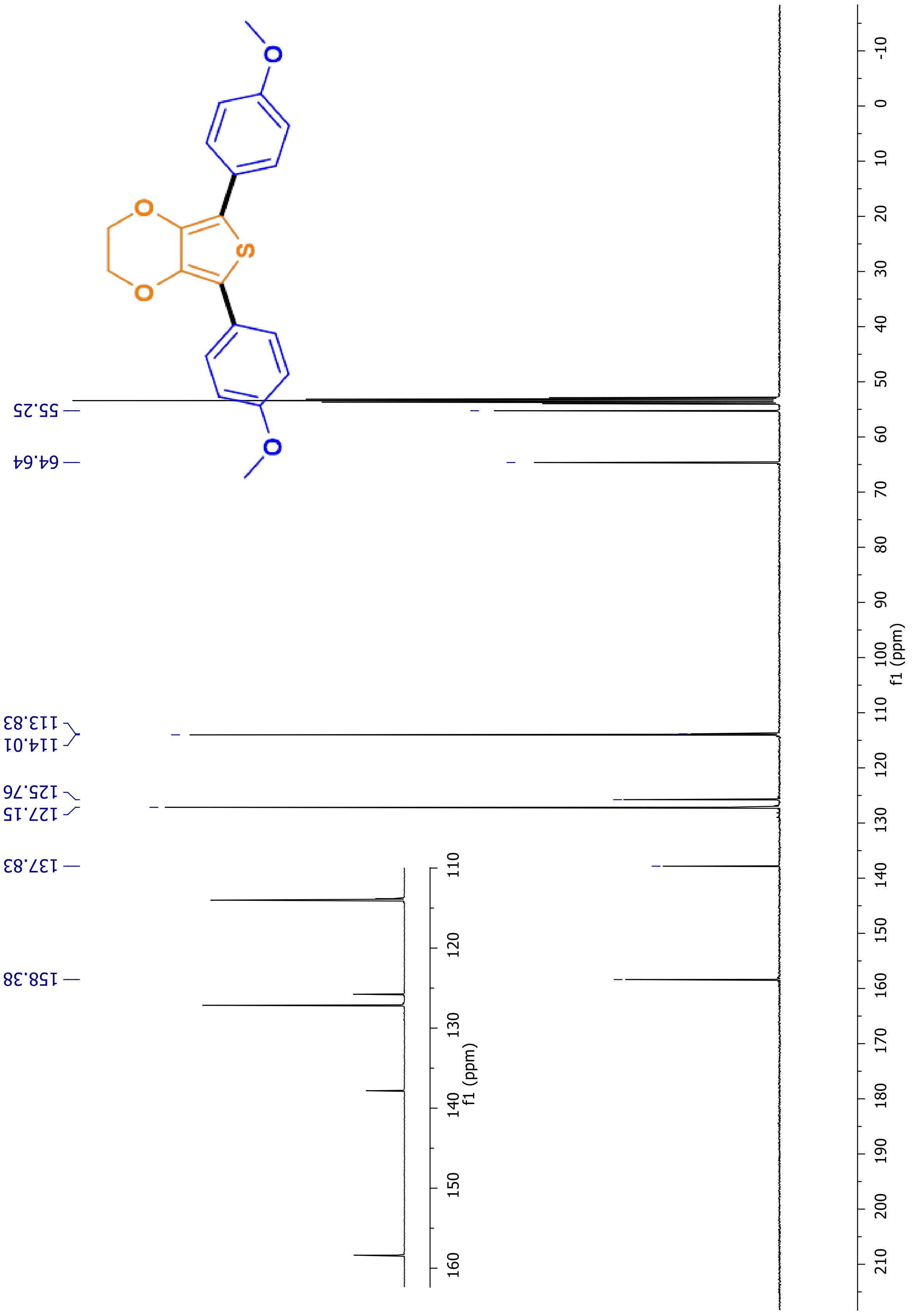

^{13}C NMR spectrum of derivative 8.

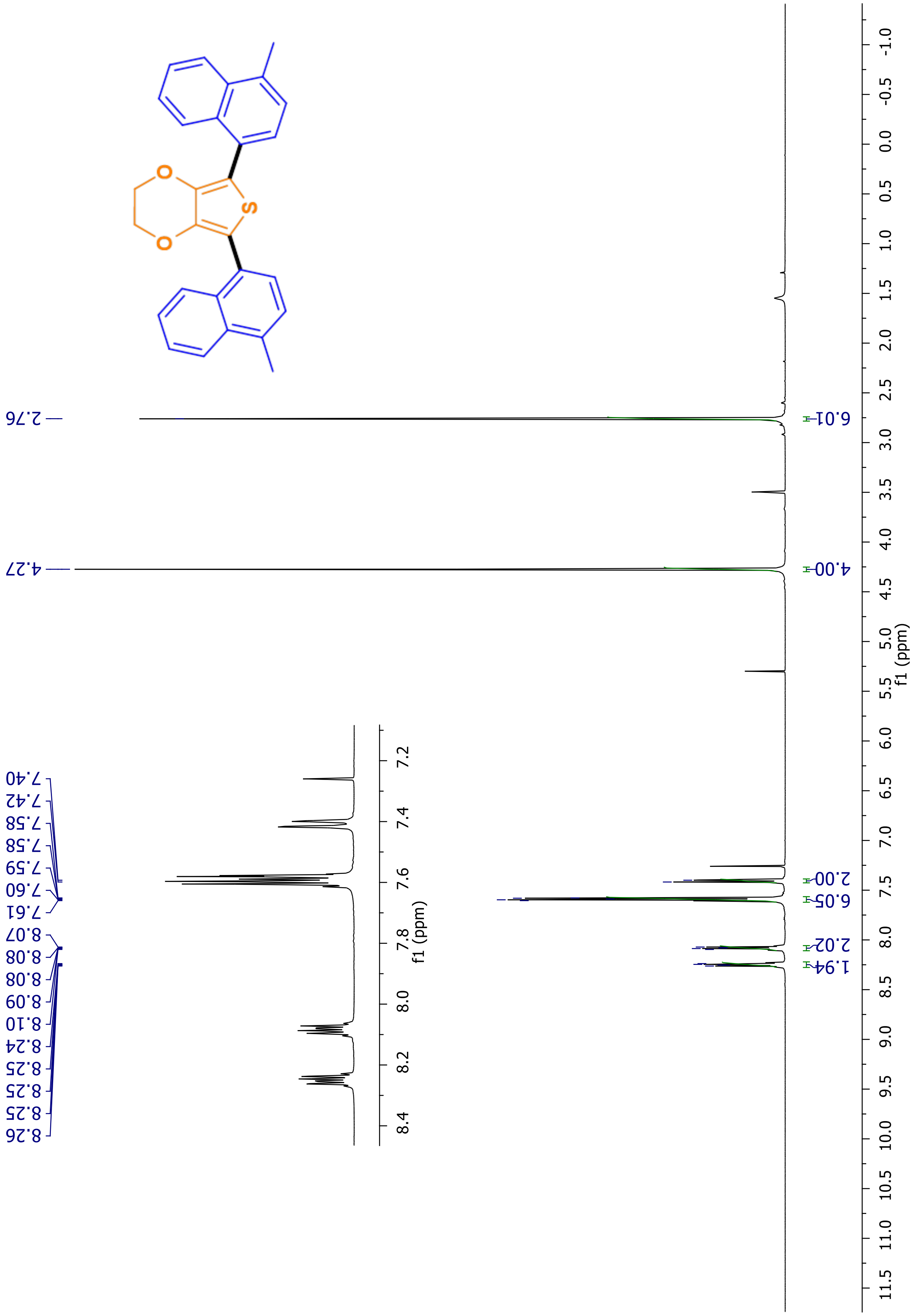

^{1}H NMR spectrum of derivative 9.

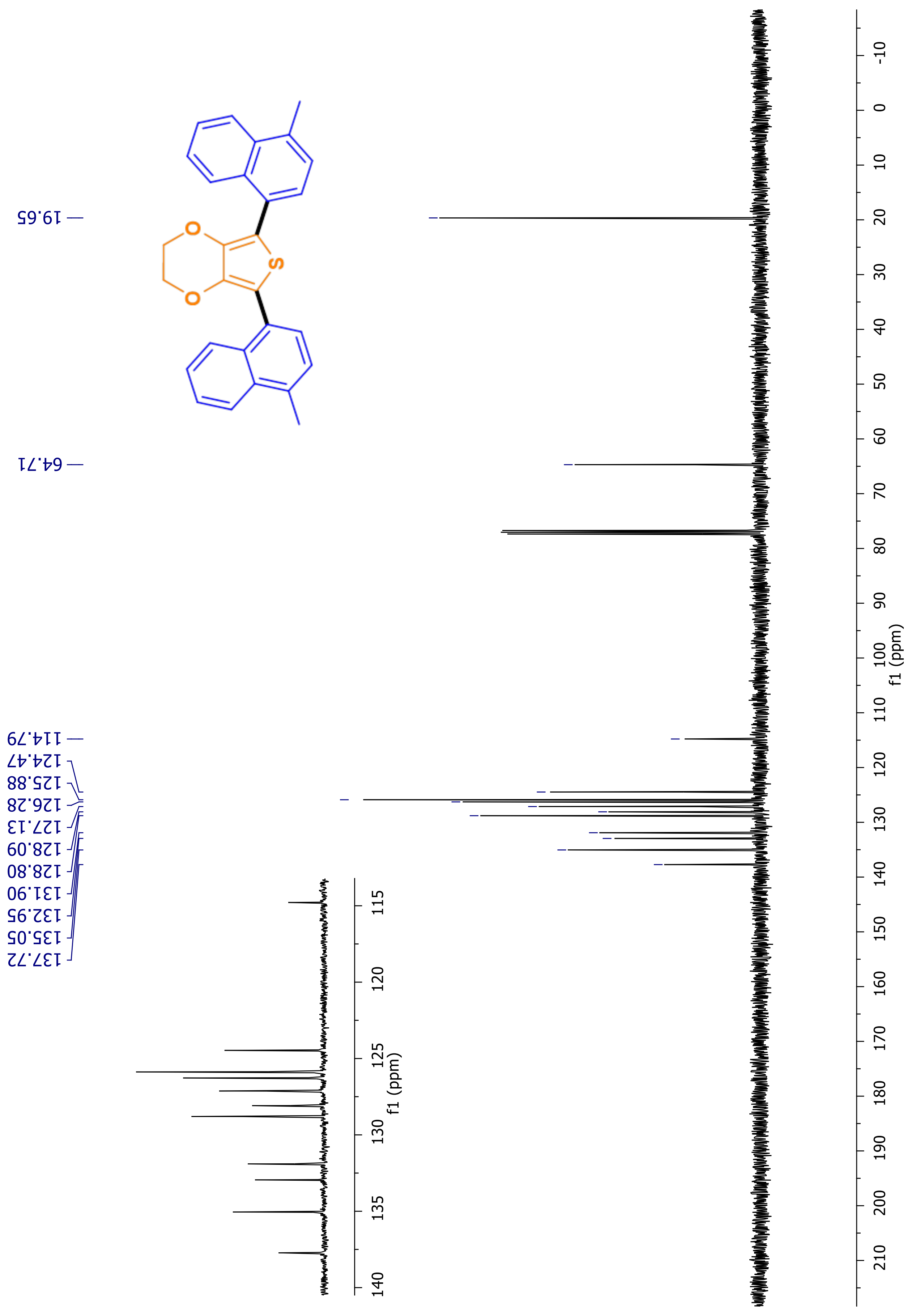

^{13}C NMR spectrum of derivative 9.

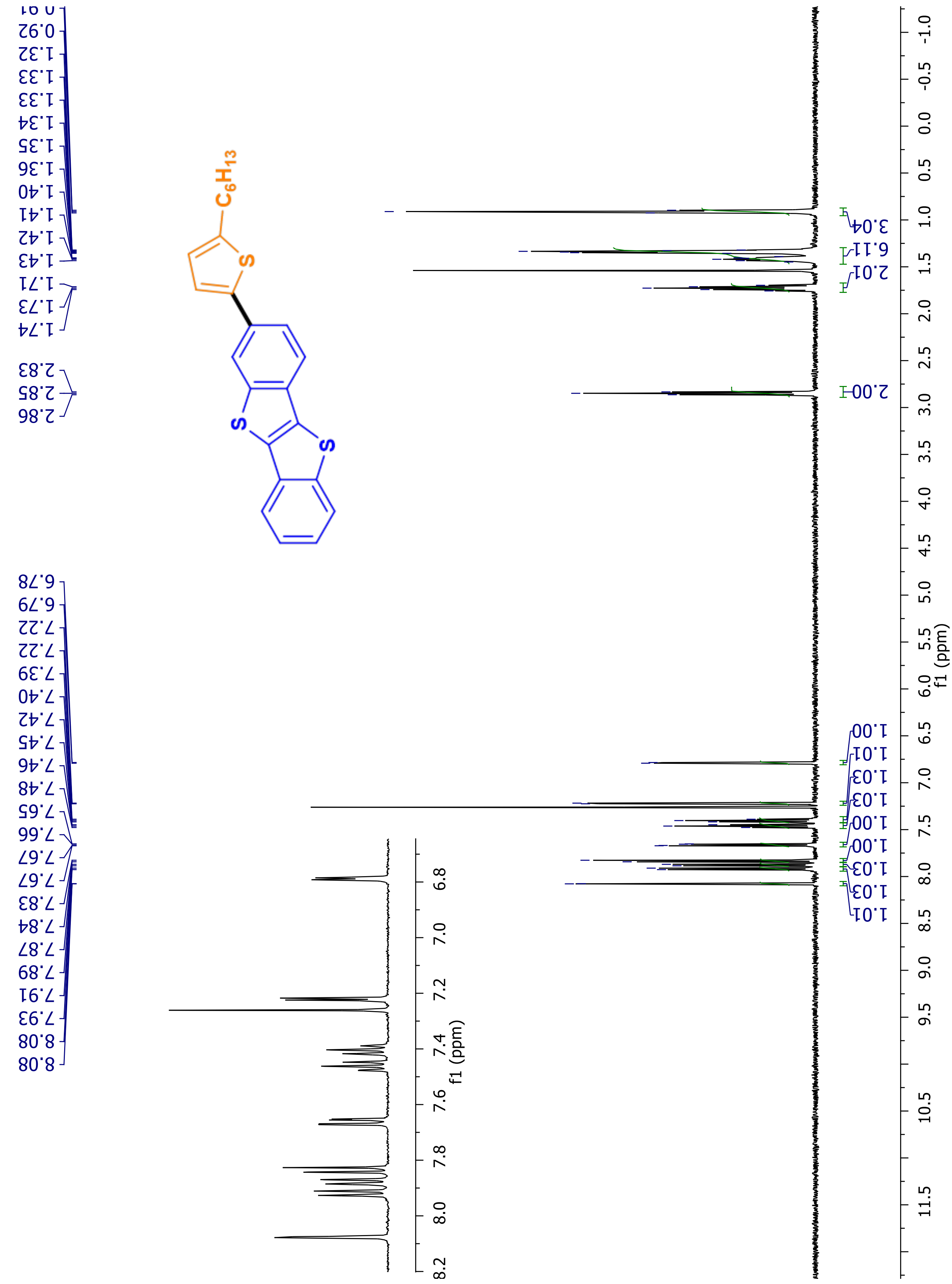

^{1}H NMR spectrum of derivative 10.

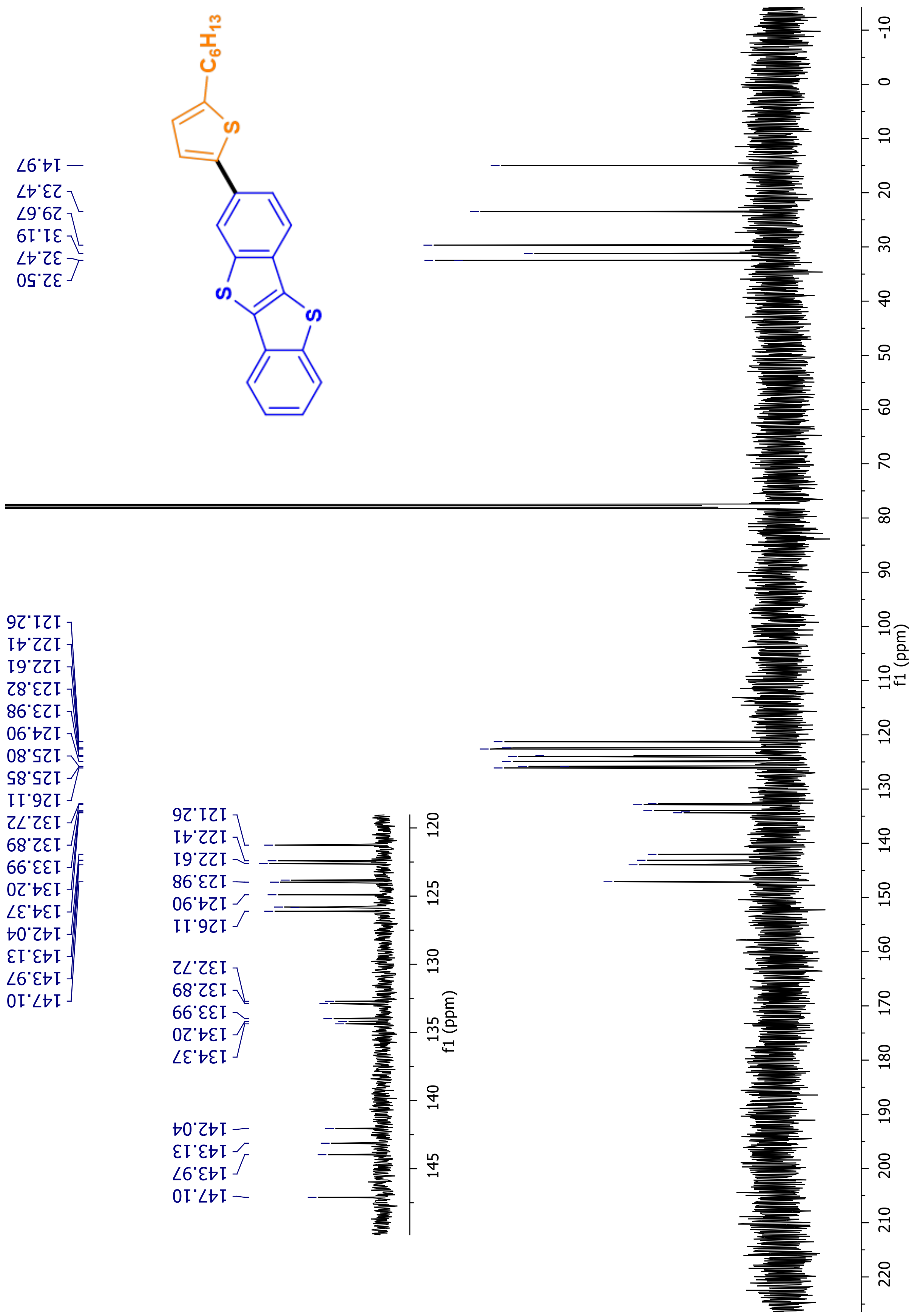

^{13}C NMR spectrum of derivative 10.

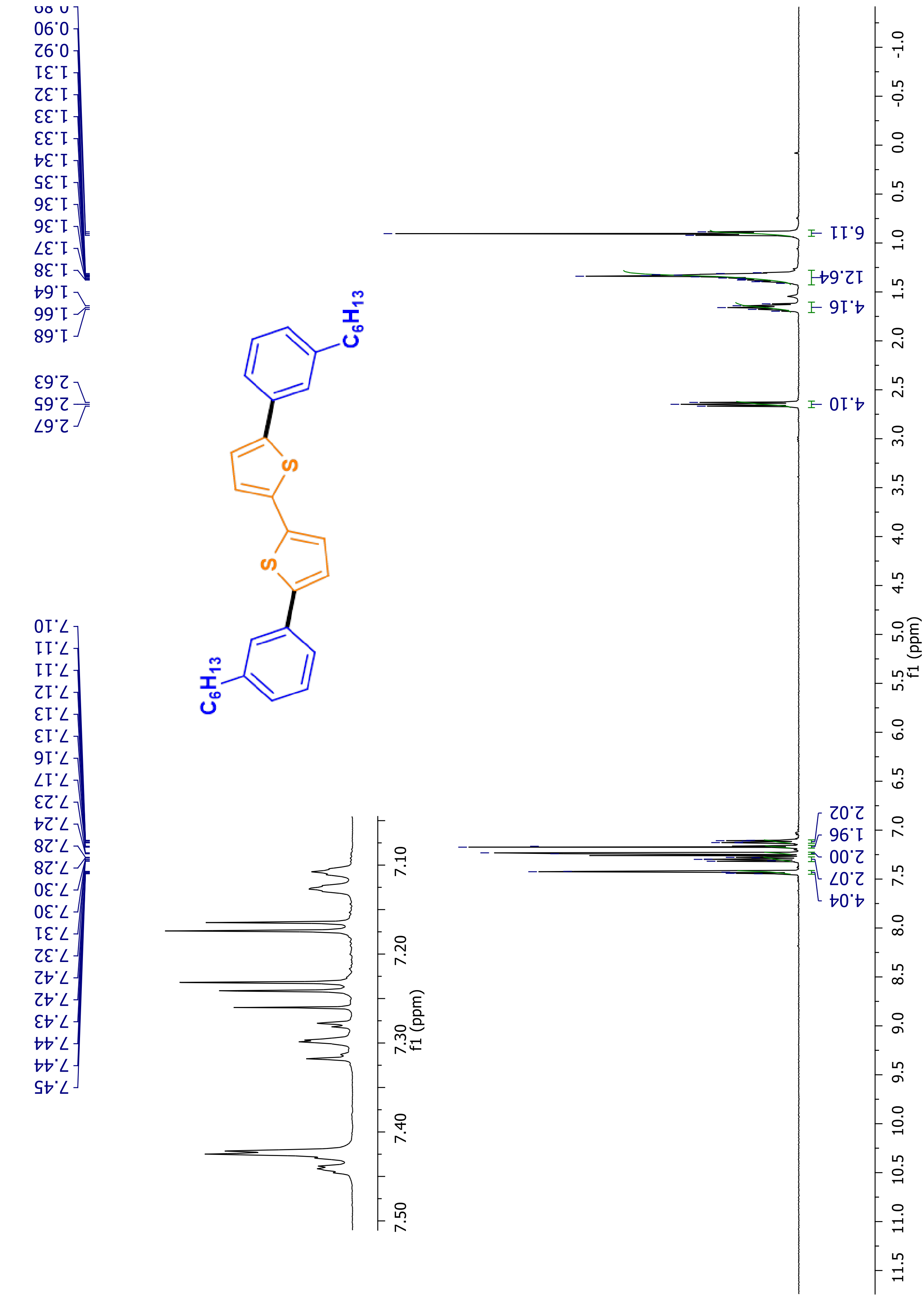

^{1}H NMR spectrum of derivative 11.

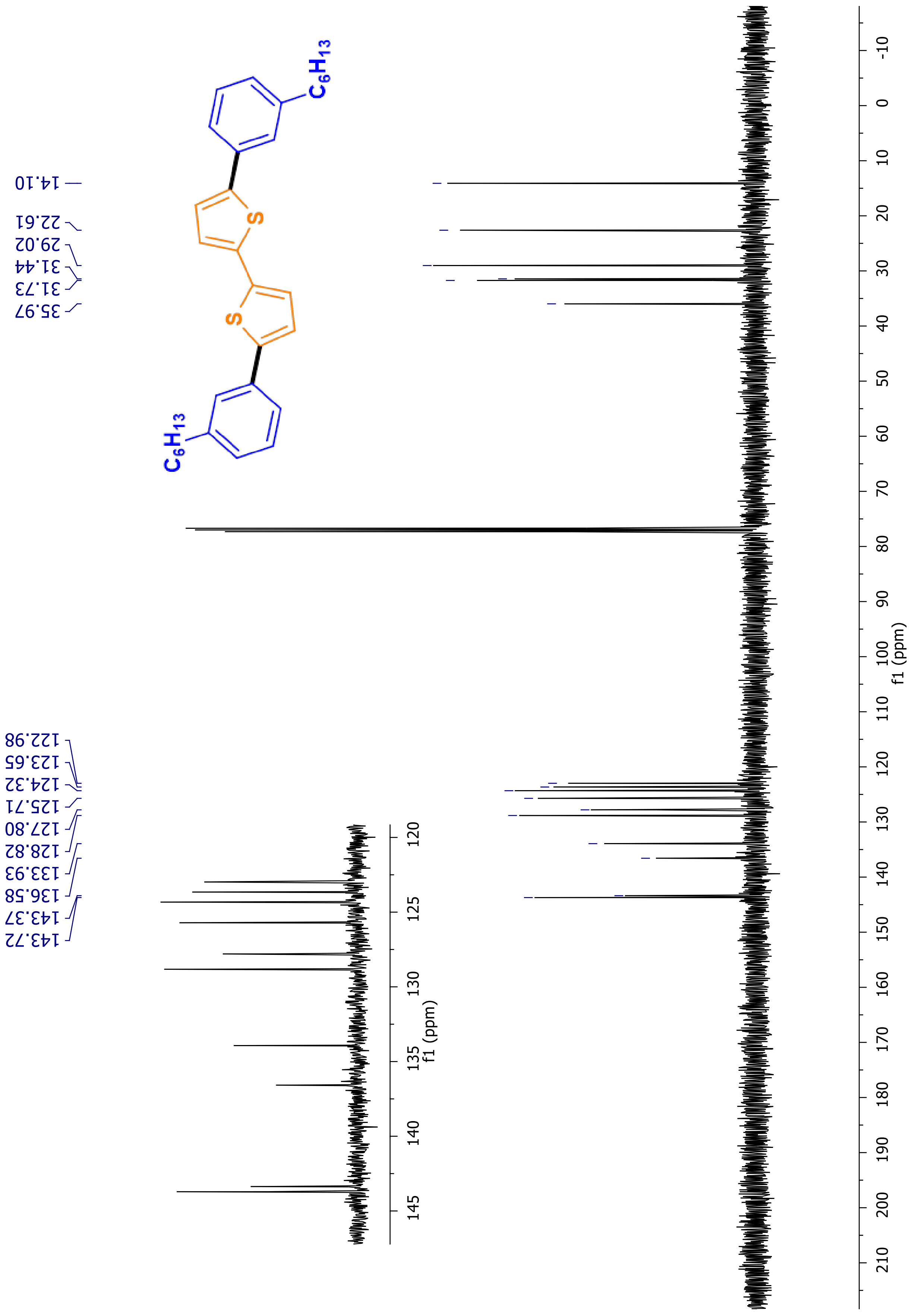

^{13}C NMR spectrum of derivative 11.

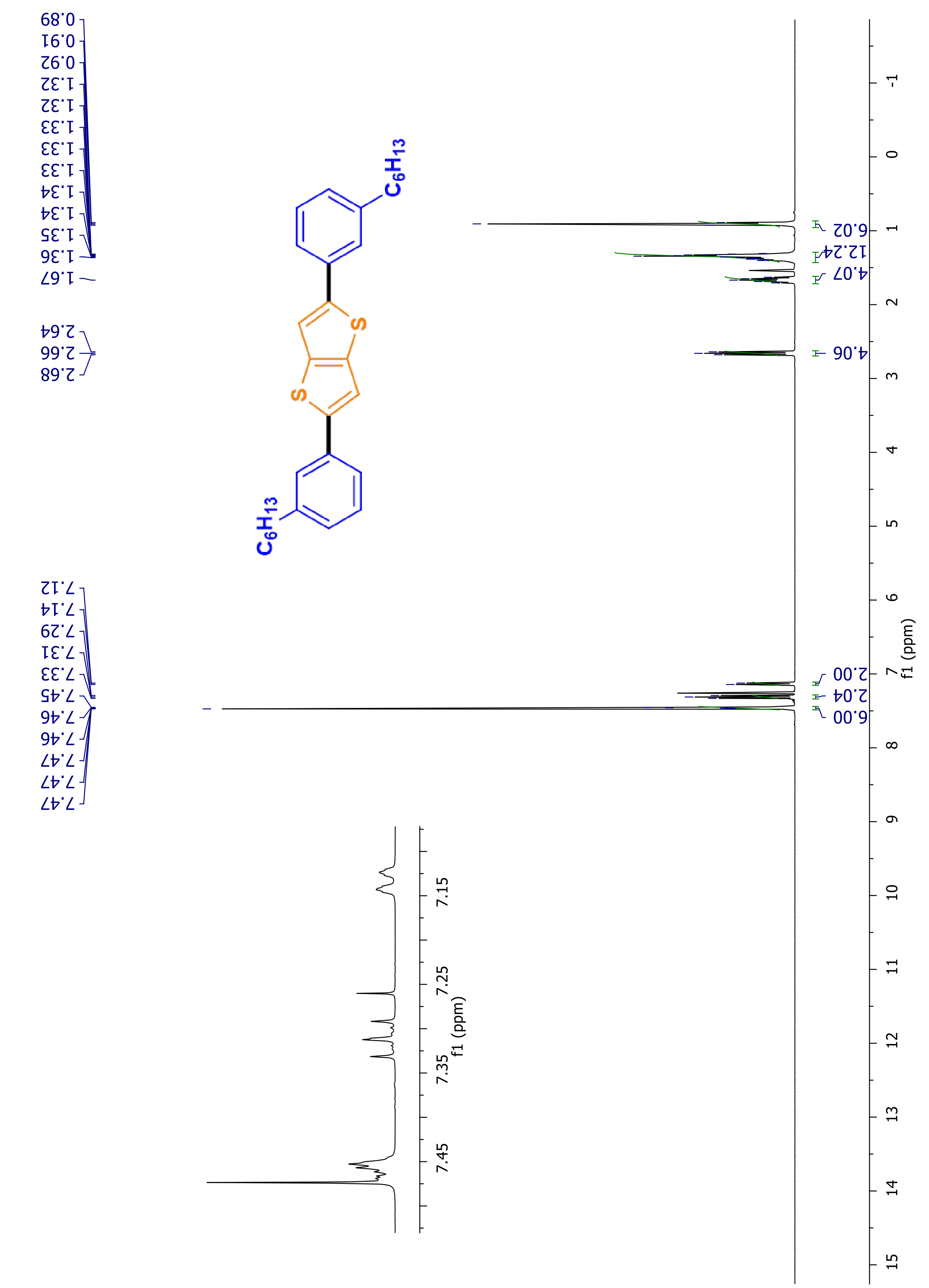

¹H NMR spectrum of derivative 12.

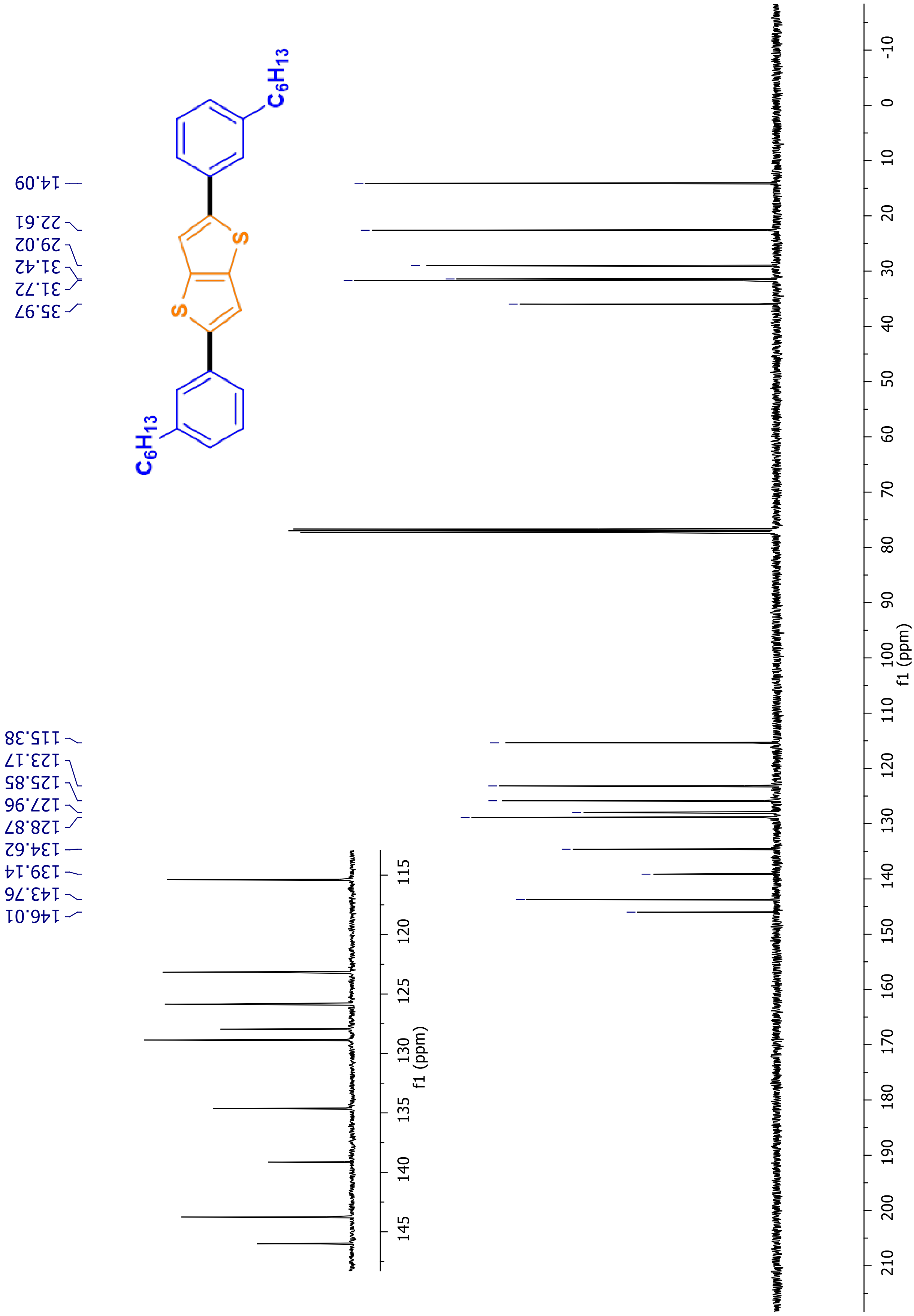

^{13}C NMR spectrum of derivative 12.

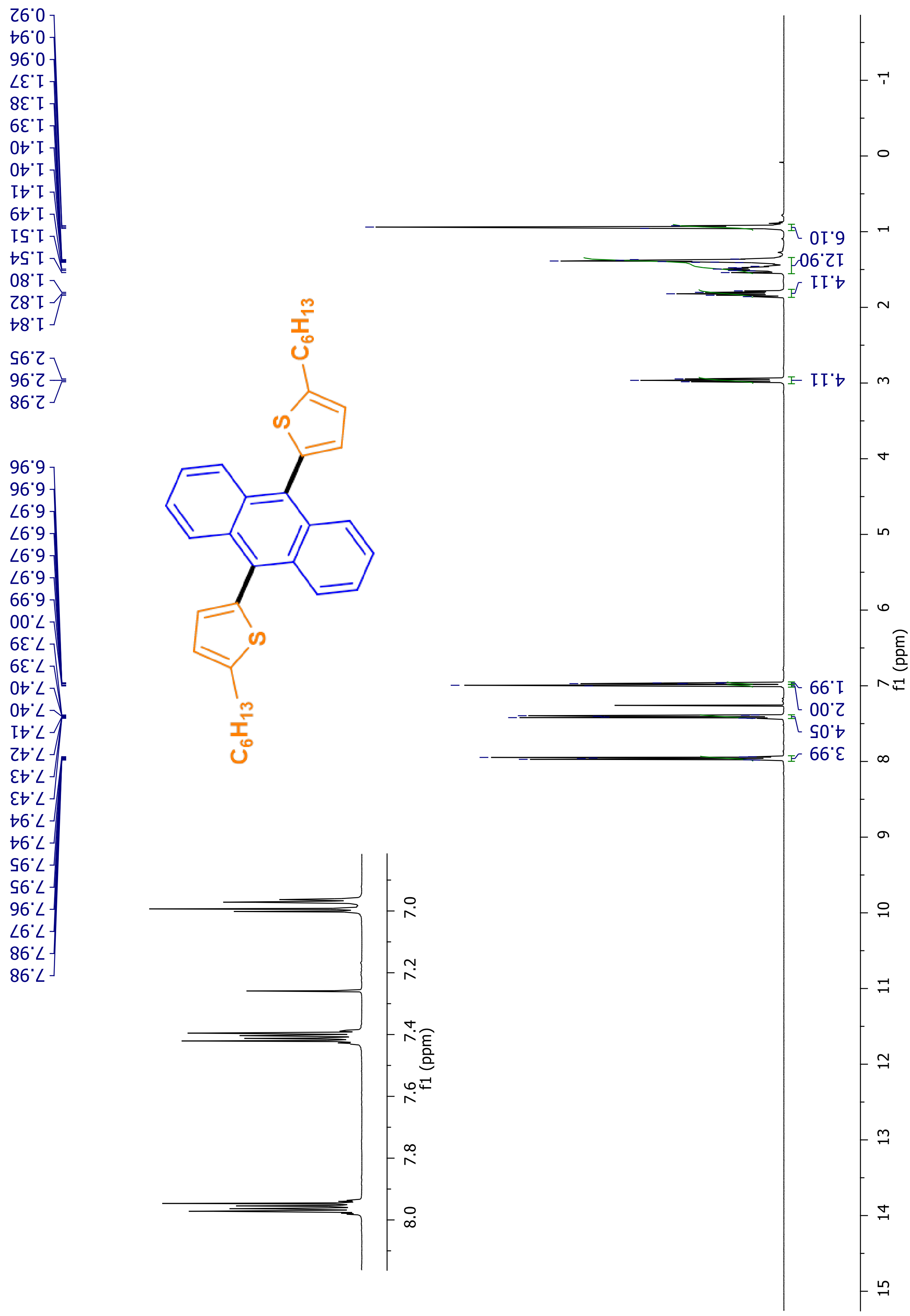

^{1}H NMR spectrum of derivative 13.

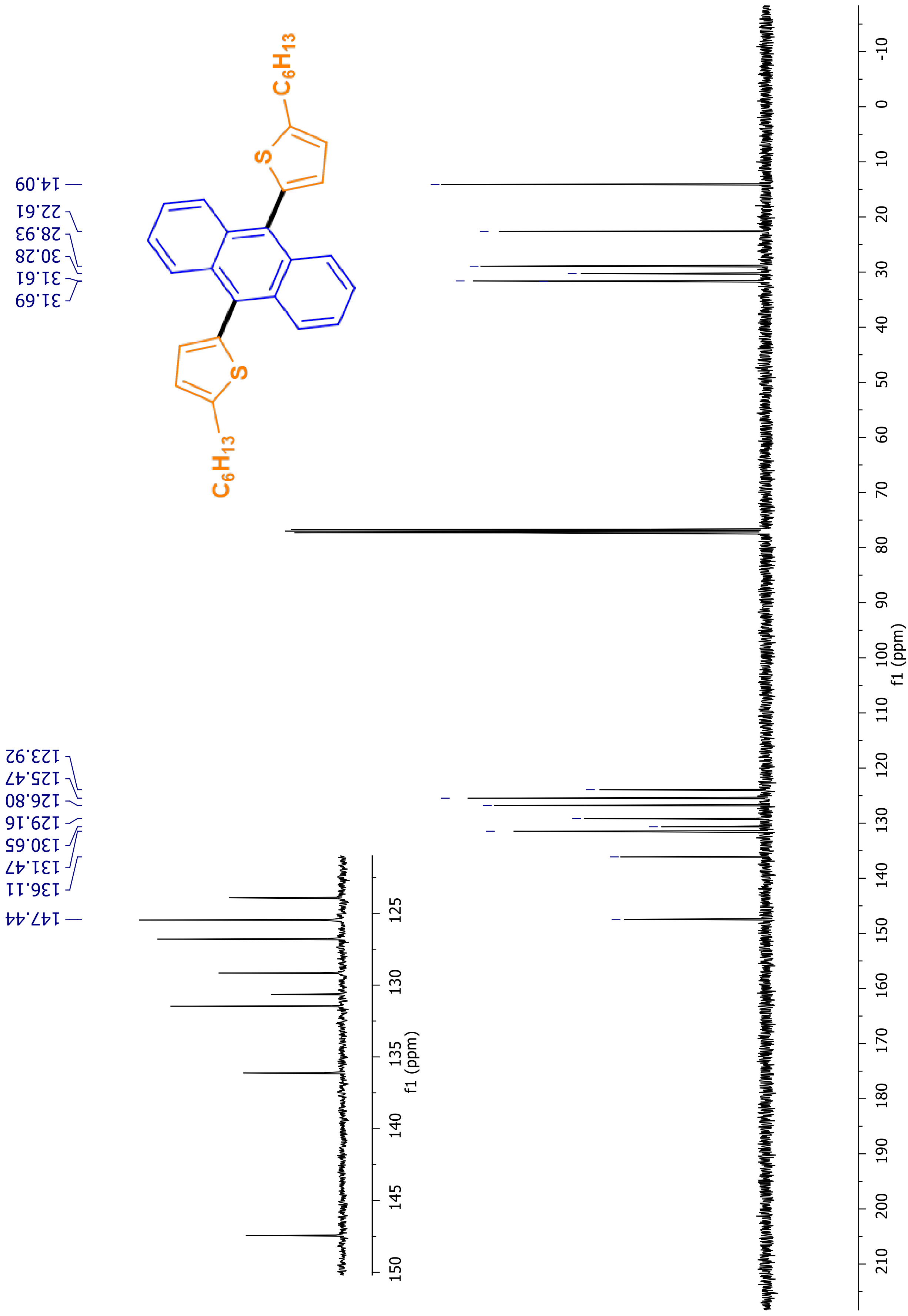

^{13}C NMR spectrum of derivative 13.

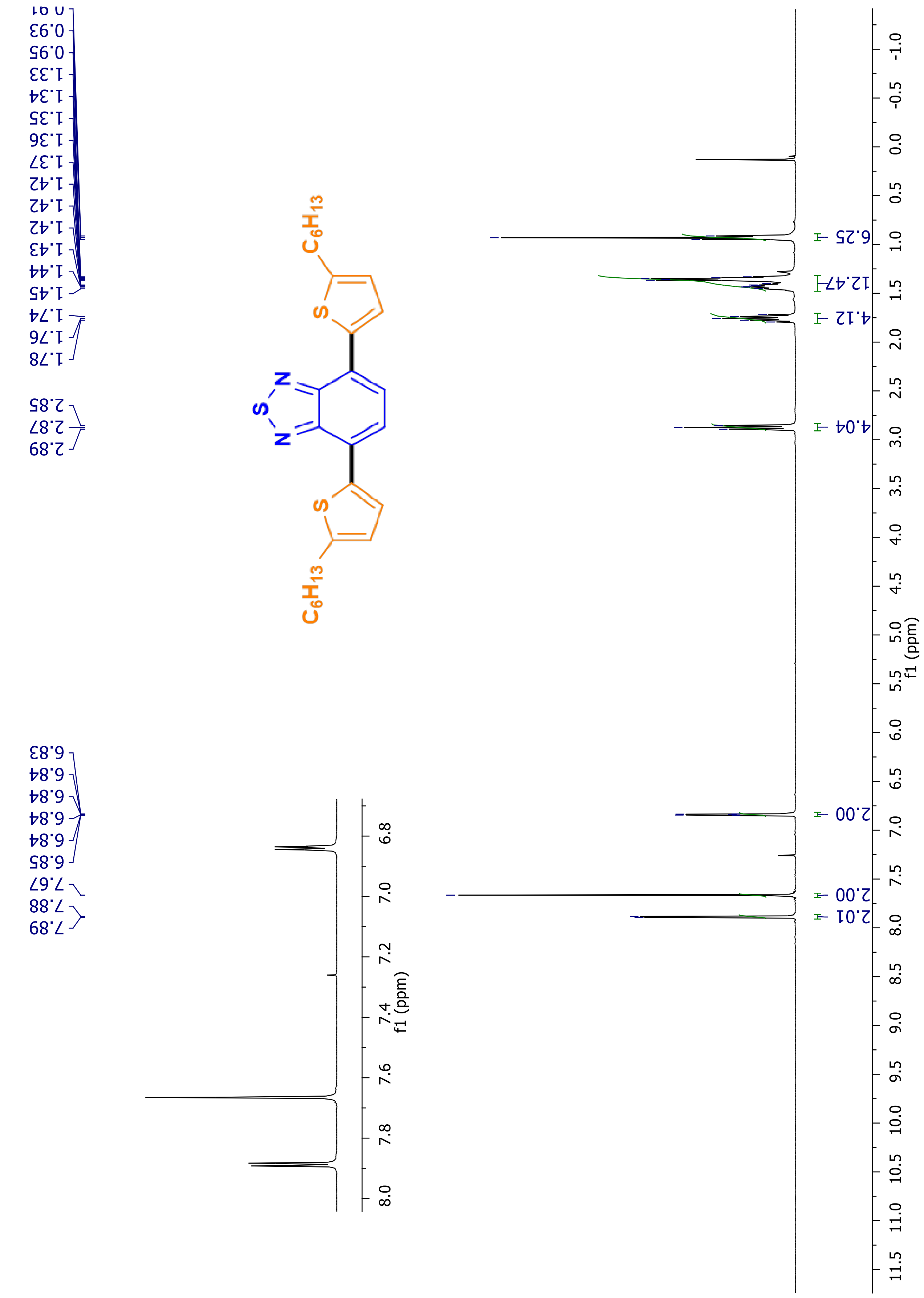

^{1}H NMR spectrum of derivative 14.

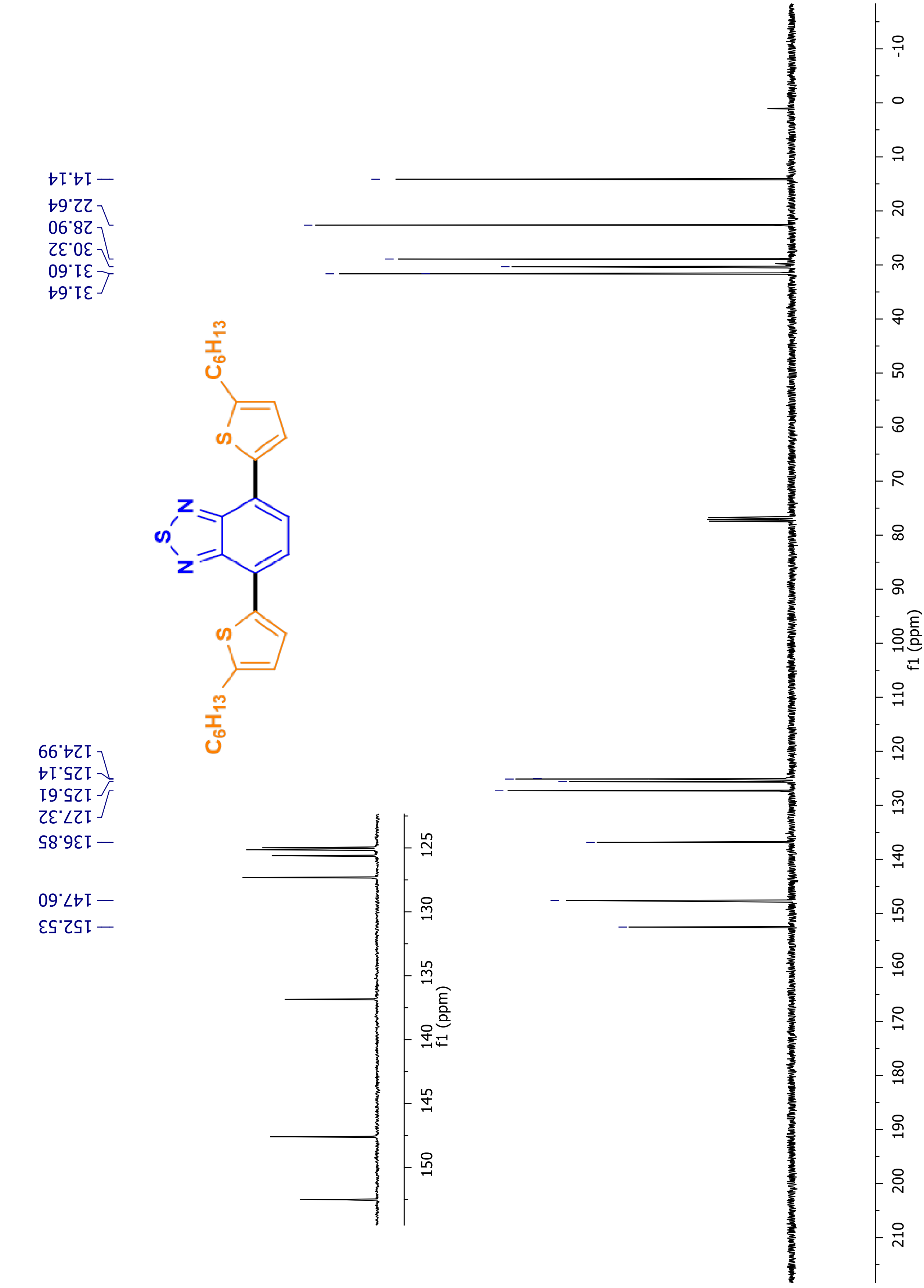

^{13}C NMR spectrum of derivative 14.

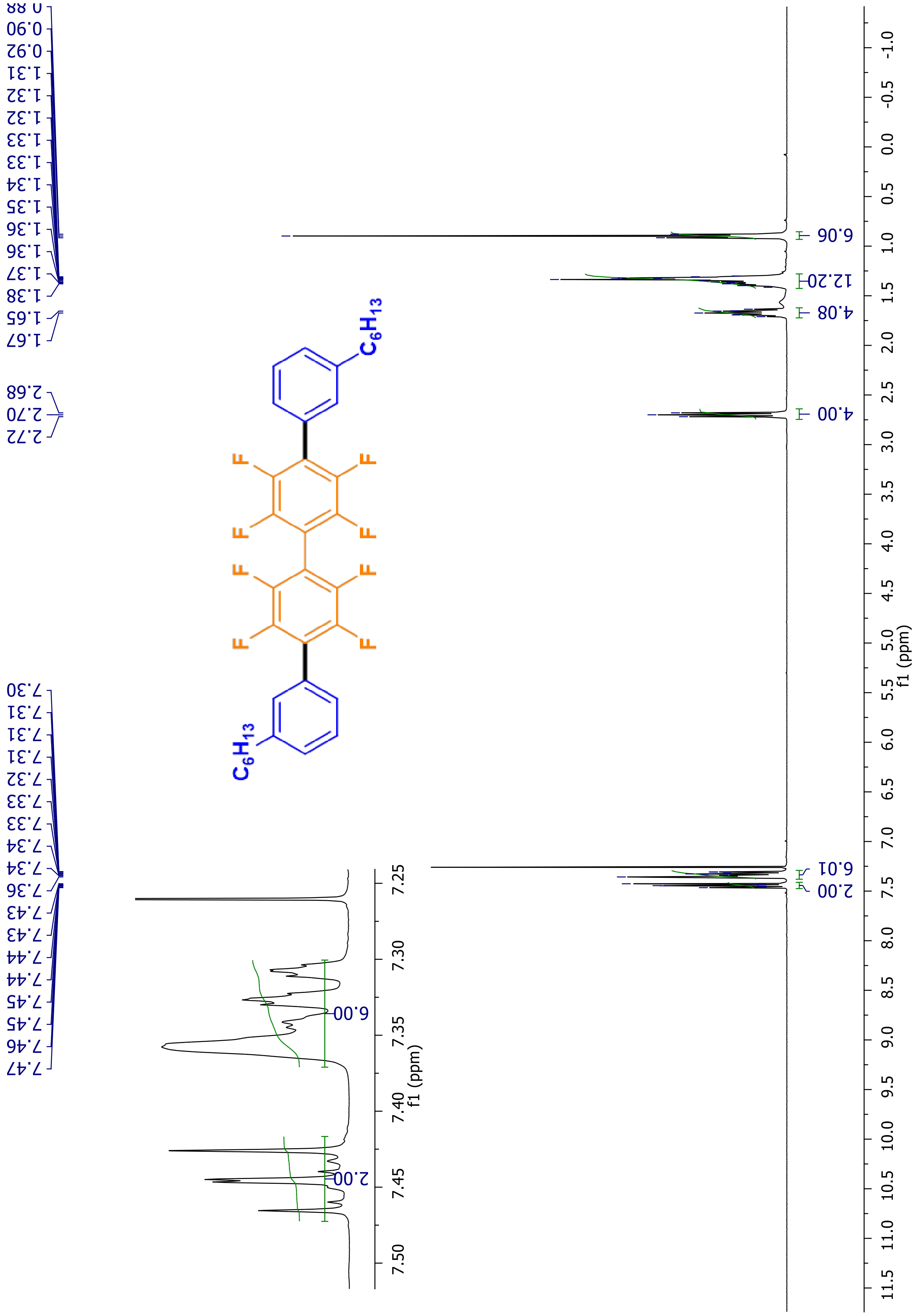

^{1}H NMR spectrum of derivative 15.

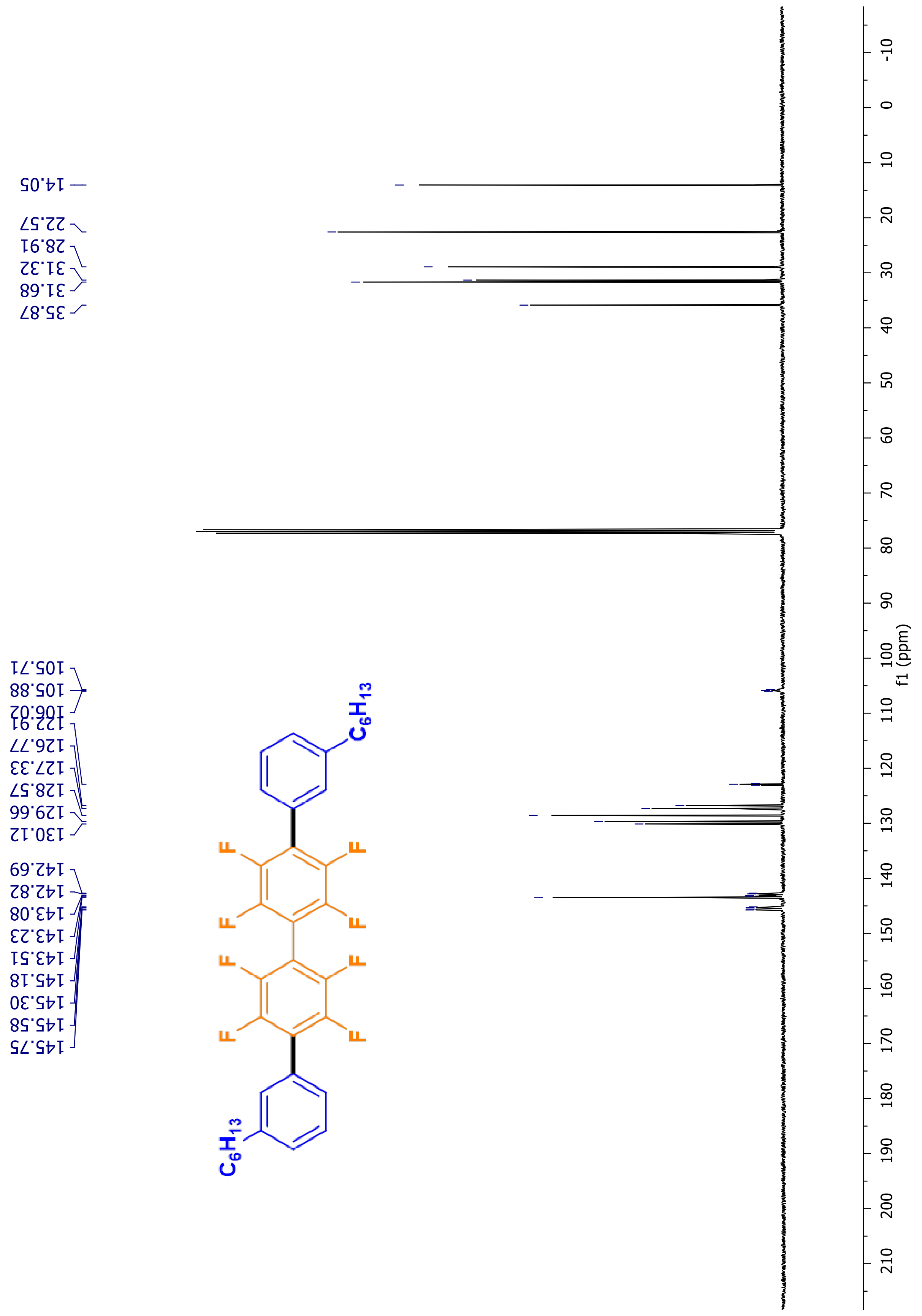

^{13}C NMR spectrum of derivative 15.
